# Amphiphilic Porphyrin Aggregates: A DFT Investigation

**DOI:** 10.3390/molecules25010133

**Published:** 2019-12-29

**Authors:** Federica Sabuzi, Manuela Stefanelli, Donato Monti, Valeria Conte, Pierluca Galloni

**Affiliations:** Department of Chemical Sciences and Technologies, University of Rome Tor Vergata, Via della Ricerca Scientifica snc, 00133 Rome, Italy; manuela.stefanelli@uniroma2.it (M.S.); monti@stc.uniroma2.it (D.M.); valeria.conte@uniroma2.it (V.C.)

**Keywords:** DFT calculations, aggregation, porphyrin dimers, cation–π dimer, H-type dimer

## Abstract

Owing to the attractive potential applications of porphyrin assemblies in photocatalysis, sensors, and material science, studies presently concerning porphyrin aggregation are widely diffused. π–π stacking, H-bonding, metal coordination, hydrophobic effect, and electrostatic forces usually drive porphyrin interaction in solution. However, theoretical studies of such phenomena are still limited. Therefore, a computational examination of the different porphyrin aggregation approaches is proposed here, taking into account amphiphilic [5-{4-(3-trimethylammonium)propyloxyphenyl}-10,15,20-triphenylporphyrin] chloride, whose aggregation behavior has been previously experimentally investigated. Different functionals have been adopted to investigate the porphyrin dimeric species, considering long-range interactions. Geometry optimization has been performed, showing that for the compound under analysis, H-type and cation–π dimers are the most favored structures that likely co-exist in aqueous solution. Of note, frontier orbital delocalization showed an interesting interaction between the porphyrin units in the dimer at the supramolecular level.

## 1. Introduction

Porphyrin assemblies are widespread in nature, where they are engaged in essential processes, such as efficient transport or storage of oxygen through the blood plasma or electron transfer in photosynthesis. Inspired by the diverse self-assembly motifs in nature, researchers have constructed and applied synthetic porphyrin systems in a variety of applicative fields, ranging from photocatalysis to sensors and material science [1,2,3,4,5,6,7,8,9,10,11,12,13,14]. Indeed, the choice of porphyrin-based building blocks to organize supramolecular systems is extremely advantageous for several reasons: (i) their chemical versatility supported by well-established synthetic methodologies; (ii) their distinctive optical features; and (iii) their invaluable photophysical and photochemical properties that enhance the assembled materials, with obvious impacts on the performance in technological applications. These chromophores can organize elaborated architectures by an assorted set of non-covalent interactions (i.e., π–π stacking, H-bonding, metal coordination, hydrophobic effect, and electrostatic forces) between the tetrapyrrolic macrocycles, mostly in J- or H-aggregated species, where aromatic platforms are stacked side by side and face to face, respectively. UV-vis spectroscopy allows the identification of the porphyrin arrangement within the aggregates, through the modified spectral features of the porphyrin monomer following the interaction among the porphyrin units. Indeed, it is well known that red-shifted UV-vis absorptions with respect to those of monomeric form are associated with J-type aggregates, while their blue shifts are distinctive of H-aggregation.

Several methods can be used to assemble porphyrin derivatives, as template-directed, surfactant-assisted, and deposition methods or pH-induced self-assembly protocol, among others [15,16,17,18,19,20,21,22,23,24,25,26]. Over the course of our studies focused on the development of amphiphilic porphyrin-based supramolecular systems, we largely used the “good–bad” solvent self-assembling method, by which macrocycles are at first molecularly dissolved in a good solvent and then a bad solvent is added to trigger the aggregation process. Operating carefully within the experimental parameters, i.e., concentration of porphyrin solutions, type, and order of addition of the two solvents and time, assemblies with different structures can be achieved [27,28]. We typically used ethanol/water or dimethylacetamide/water solvent combinations at proper water contents to drive the formation of ordered porphyrin suprastructures by hydrophobic effect [21,29,30,31,32].

Despite plenty of experimental investigations dealing with the self-organization of porphyrin both in solution and at the solid state [33,34,35], computational studies have lagged behind.

**Figure 1:**
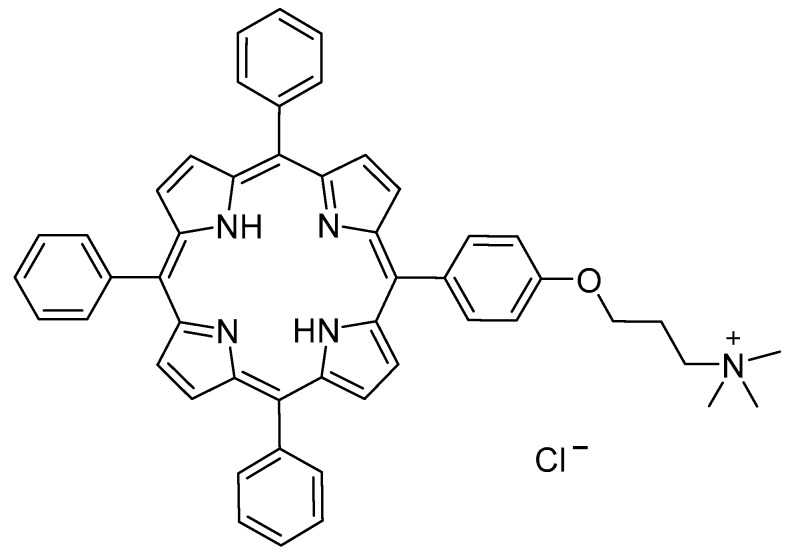
Structure of **1H_2_**.

Therefore, the aim of this work is to define a appropriate computation approach to study and characterize porphyrin aggregates, to get more insights into the kind of the interactions responsible for the formation of specific non-covalent systems (J-, H- or different aggregates) experimentally observed. Herein, the computationally investigated phenomenon is the solvent-driven aggregation of the amphiphilic porphyrin derivative [5-{4-(3-trimethylammonium)propyloxyphenyl}-10,15,20-triphenylporphyrin] chloride (**1H_2_**) (Figure 1) in water–ethanol (9:1 *v/v*) solvent mixture, as reported before [36].

## 2. Results and Discussion

**Figure 2:**
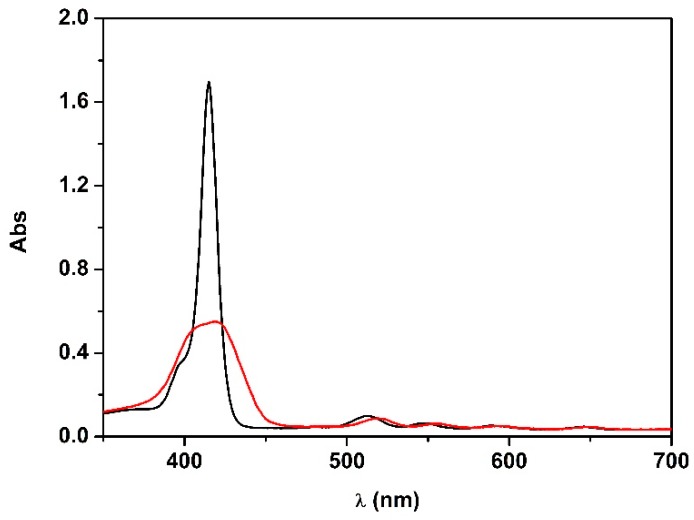
UV-vis spectra of **1H_2_** (4.0 × 10^−6^ M) in EtOH (black line) and in EtOH:H_2_O 1:9 *v/v* (red line).

[5-{4-(3-trimethylammonium)propyloxyphenyl}-10,15,20-triphenylporphyrin] chloride (**1H_2_**) was synthesized according to a previously reported procedure [36]. UV-visible spectrum of **1H_2_** in ethanol showed a sharp and intense Soret band centered at 415 nm, and four Q-bands between 500 and 700 nm, characteristic of the monomeric species. Experimental studies showed that upon solvent change from ethanol to a mixture of ethanol–water 1:9 *v/v*, porphyrin aggregation was induced. In particular, the gradual increase of the water content in the mixture led to the broadening of the Soret band, as well as the lowering of the molar extinction coefficient (Figure 2) [36]. Such effects are typically correlated with the formation of aggregates in solution.

To understand the nature of such aggregates, DFT calculations were performed. The flat and electron-rich surface of the porphyrins promotes self-aggregation through van der Waals, π–π, and charge-transfer interactions, leading to the well-known H- or J-type aggregates [37]. Importantly, the amphiphilic nature of **1H_2_** also makes feasible cation–π interactions [38], since the positive-charged chain, bound at the *para*- position of the phenyl group, can interact with the electron-rich porphyrin core.

Therefore, geometry optimization of the monomeric porphyrin and the possible aggregated species has been carried out.

**1H_2_** structure has been optimized both in the vacuum and in ethanol, using the WB97XD functional and 6-31G(d) basis set. Recently, an excellent agreement with experimental data has been achieved using the same functional for other cationic porphyrins [39]. The solvent effect has been included using the polarizable continuum model (PCM).

DFT calculations confirmed the flat geometry of the porphyrin core, while phenyl groups at the meso positions were located edgeways (Figure S1).

**Figure 3:**
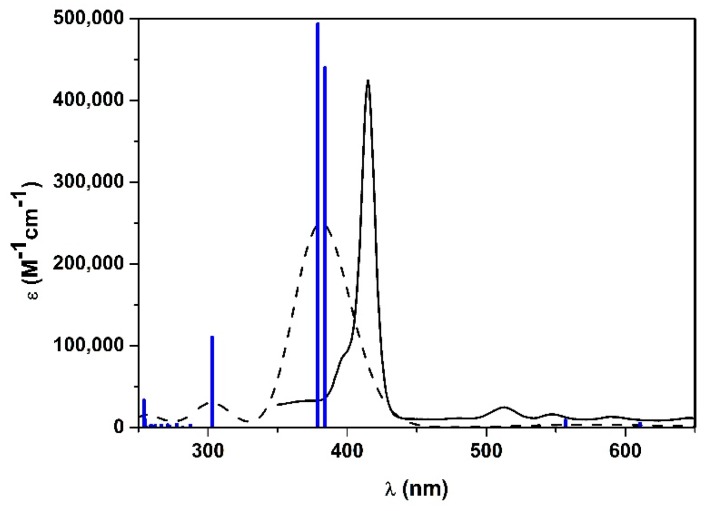
Experimental UV-vis spectrum of **1H_2_** in EtOH (black line), calculated spectrum with WB97XD functional and 6-311G(d,p) basis set (black-dashed line) and the corresponding calculated electronic transitions (blue bars).

UV-vis spectrum as well as the theoretical electronic transitions were predicted using TD-DFT, with the same functional and 6-311G(d,p) basis set. The first 20 transition electronic states have been calculated. The experimental spectrum in EtOH [36] was compared with the calculated one (Figure 3). The simulated absorption spectrum correctly followed the trend of the experimental one, but it resulted in being broader and was highly blue-shifted in the Soret band region (λ_max-WB97XD_ = 382 nm; λ_max-exp_ = 415 nm). Q-bands were represented as a unique band with very low intensity, which is a common feature for such class of compounds, because of the small theoretical oscillator strength at those wavelengths [39,40,41].

**Figure 4:**
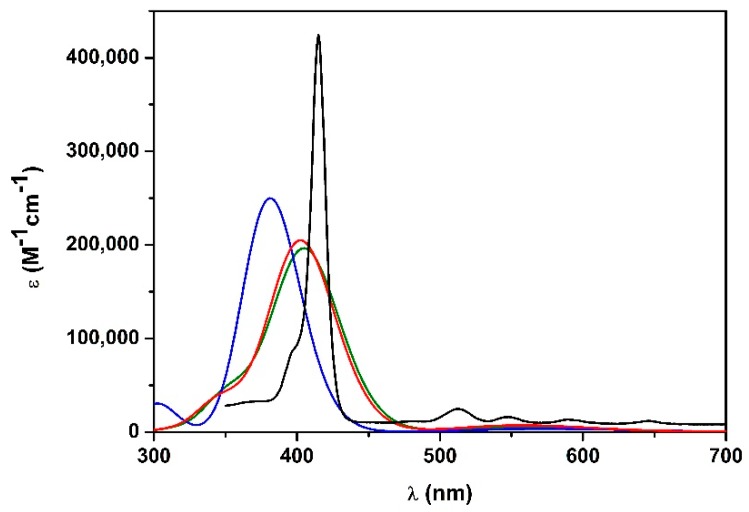
Calculated UV-vis spectra in EtOH of **1H_2_** with: B3LYP functional 6-311G(d,p) basis set (green line), CAM-B3LYP functional 6-311G(d,p) basis set (red line), WB97XD functional 6-311G(d,p) basis set (blue line). Experimental UV-vis spectrum of **1H_2_** in EtOH is represented with the black line.

Hence, some attempts to improve the calculated spectrum have been accomplished using different functionals (Figure 4). Notably, CAM-B3LYP (which also includes the long-range interaction correction) previously gave excellent results with similar positive-charged porphyrin [39]. Thus, geometry optimization has been performed with B3LYP (a conventional functional) and CAM-B3LYP, using 6-31G(d) basis set. Afterwards, TD-DFT with the corresponding functionals and 6-311G(d,p) basis set has been performed. In both cases, the Soret band gave a broad but much more similar result to the experimental one (λ_max-CAM-B3LYP_ = 403 nm; λ_B3LYP_ = 405 nm; λ_max-exp_ = 415 nm) and Q-bands were more intense than those obtained with WB97XD (Figure 4).

Results revealed that B3LYP and CAM-B3LYP better represent the absorption spectrum of **1H_2_** in EtOH. Therefore, it can be assumed that WB97XD functional is suitable when a positive charge is delocalized over a π system, as previously observed [39,42]. Conversely, if the cation is located at the terminal position of a saturated alkyl side chain, and there is no interaction with the π-core, as in **1H_2_** porphyrin, conventional functionals can be adopted.

**Figure 5:**
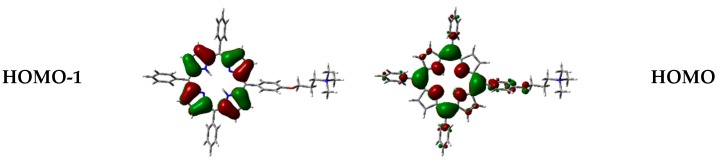

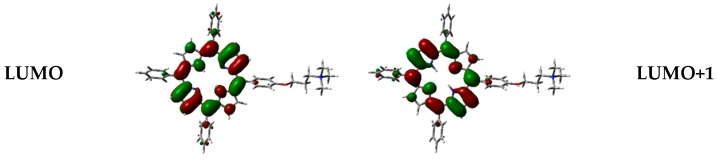
Frontier orbitals of **1H_2_** calculated with B3LYP functional 6-31G(d) basis set.

On this basis, B3LYP has also been chosen to visualize frontier orbitals. As typically observed for porphyrinoid systems, orbitals are delocalized over the porphyrin macrocycle (Figure 5), being responsible for π→π* transitions [39,40,41], while the positive-charged chain is not involved.

**Figure 6:**
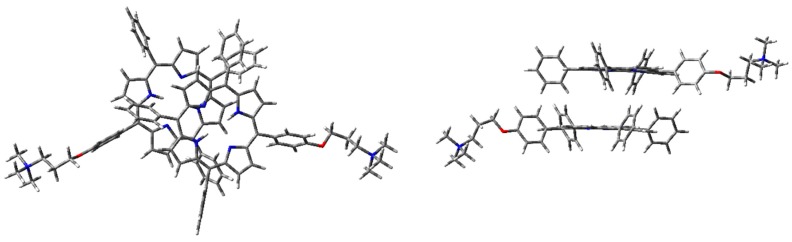
**1H_2_** H-type dimer geometry optimization in the vacuum with CAM-B3LYP functional and 6-31G(d) basis set: front (**left**) and side (**right**) view.

**Figure 7:**
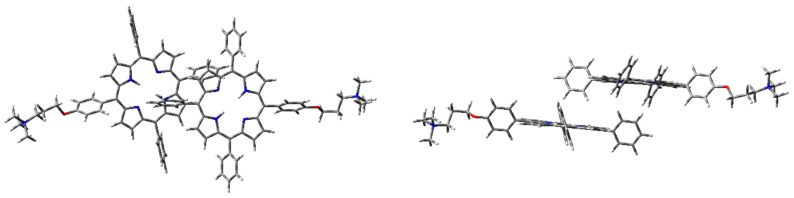
**1H_2_** J-type dimer geometry optimization in the vacuum with CAM-B3LYP functional and 6-31G(d) basis set: front (**left**) and side (**right**) view.

**Figure 8:**
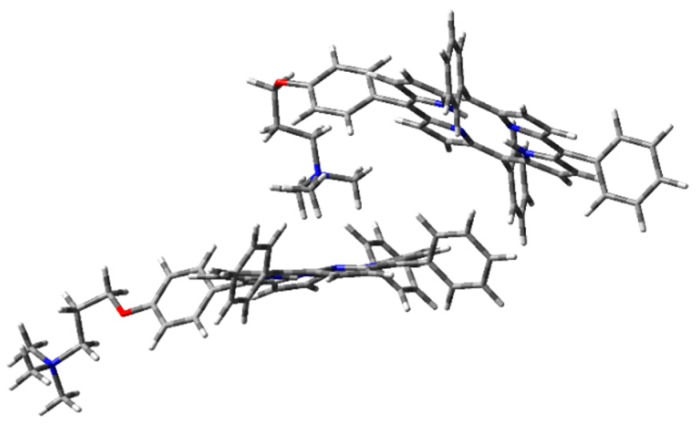
**1H_2_** cation–π dimer geometry optimization in the vacuum with CAM-B3LYP functional and 6-31G(d) basis set.

Aggregation studies have been carried out, considering the simplest supramolecular structure, namely the dimer. Therefore, H-, J-, and cation–π dimers of **1H_2_** have been modeled. Although B3LYP gave good results for the monomer in EtOH, in the case of the dimers CAM-B3LYP functionality has been adopted for geometry optimizations, since it considers long-range interactions (that are fundamental for intermolecular systems). Furthermore, good predictions were achieved for the electronic absorption spectrum of the monomer. In the case of the dimer, correction for basis set superposition error (BSSE) has been applied, using the counterpoise correction (CP) [43]. Thus, dimer geometry optimization was initially performed with CAM-B3LYP functional and 6-31G(d) basis set, in the vacuum. Optimized geometry of the dimers is presented in Figure 6, Figure 7 and Figure 8.

The optimized H-type aggregate converged to a partially differently organized structure, where the two porphyrin cores were slightly staggered to each other (Figure 6). The positive-charged substituents were located at the opposite sides of the molecules, to reduce charge repulsion. Moreover, the porphyrin cores appeared slightly rotated, one with respect to the other, to reduce steric hindrance of the edgeways phenyl groups. The calculated interplanar distance between the porphyrin macrocycles was nearly 5.4 Å. Such value was quite high for π–π-stacked systems, where the distance between two parallel molecular planes is usually in the range 3.3–3.8 Å [44]. Similarly, the optimization of the modeled J-type dimer led to two almost non-interacting units, where porphyrin planes were ca. 5.0 Å apart (Figure 7). Notably, among the others, the cation–π dimer was the most stable one, showing an energy value in the vacuum that was 5.7 kcal·mol^−1^ lower than the modeled H-type dimer and 3.5 kcal∙mol^-1^ more stable than the optimized J-type dimeric structure (Table 1). In the cation–π dimer, the terminal ammonium group interacts with the electron-rich macrocycle, leading to a folded and stable structure (Figure 8).

**Figure 9:**
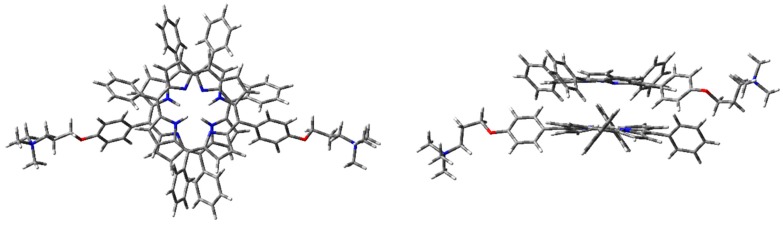
**1H_2_** H-dimer geometry optimization in the vacuum with WB97XD functional and 6-31G(d) basis set: front (**left**) and side view (**right**).

**Figure 10:**
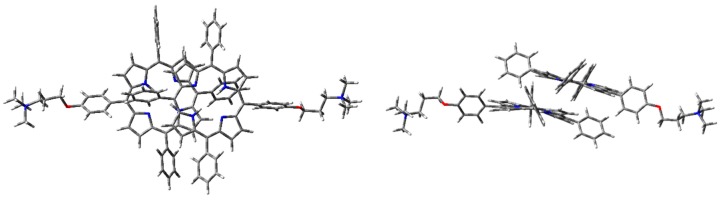
**1H_2_** J-dimer geometry optimization in the vacuum with WB97XD functional and 6-31G(d) basis set: front (**left**) and side view (**right**).

**Figure 11:**
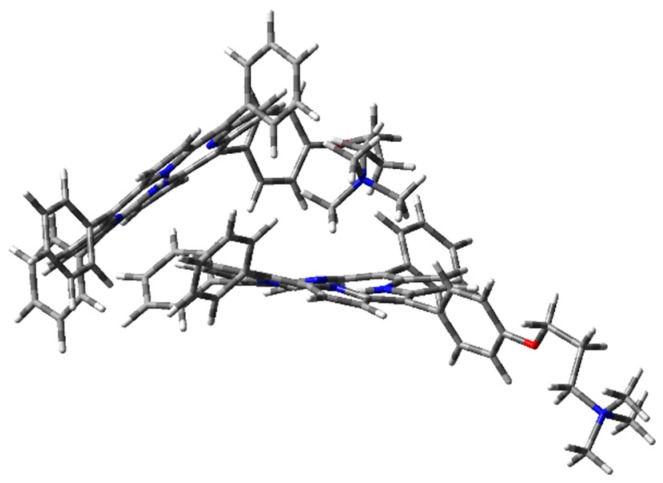
**1H_2_** cation–π dimer geometry optimization in the vacuum (PCM) with WB97XD functional and 6-31G(d) basis set.

However, due to the unexpected results obtained in the optimization of H- and J-type dimers, a more accurate analysis was needed. Therefore, the same geometry optimization in the vacuum has been performed using WB97XD functional and 6-31G(d) basis set (Figure 9, Figure 10 and Figure 11), considering counterpoise correction. Long-range interactions, as well as the empirical dispersion term of van der Waals, are included in such functionals, and both constitute significant contributions in the energy calculation of π–π stacked dimers and cationic species [39,45,46].

Using WB97XD functionals, well-organized structures were achieved. In fact, the optimized H-type aggregate showed the classical stacked structure, with the two interacting cores placed at a proper distance to allow π–π interactions (ca. 3.7 Å). Similarly, the J-type dimer converged to an ordered aggregate, with two staggered porphyrin units showing the same average distance of the H-aggregate. One of the porphyrin moieties showed a flat core, while the other was slightly tilted and the substituted phenyl groups were located at opposite sides. Again, the cation–π dimer resulted in the most stable species, with an energy value much lower than H- and J-dimers (Table 1).

In both cases, geometry optimization in the vacuum showed that the cation–π dimer was the most stable one. Excluding solvent contribution, the structure stabilization originating from the interaction between the positive-charged ammonium cation and the porphyrin core was prevalent. The same interaction was missing in the other dimeric structures, where the positive charge was localized in the ammonium group, without any stabilization. However, the effect of a polar protic solvent, such as H_2_O, can importantly influence aggregation behavior in the solution. It is worth noting that aggregation was experimentally induced upon addition of water to an ethanol solution of **1H_2_** [36]. Therefore, starting from the optimized structures in the vacuum, single-point energy calculations were performed, using water as the solvent in PCM (Table 2). Importantly, due to inconsistent results obtained in geometry optimization of H- and J-dimers, CAM-B3LYP functional was excluded from such calculations.

As expected, water contribution strongly affects dimer energy. In particular, in the H-type dimer, water solvation of the ammonium groups on the sidechain conferred high stabilization. Accordingly, the H-aggregate was the most stable in water. Conversely, considering cation–π dimers, which were the preferred geometry in the vacuum, water molecules can interact with the ammonium group, thus preventing the interaction between the cation and the porphyrin macrocycle. However, the energy difference between H- and cation–π dimers was negligible (~0.2 kcal/mol); therefore, the co-presence of both structures can be expected in the solution. Notably, even though water content also stabilized the ammonium groups in the J-type dimer, it still was energetically disfavored.

**Figure 12:**
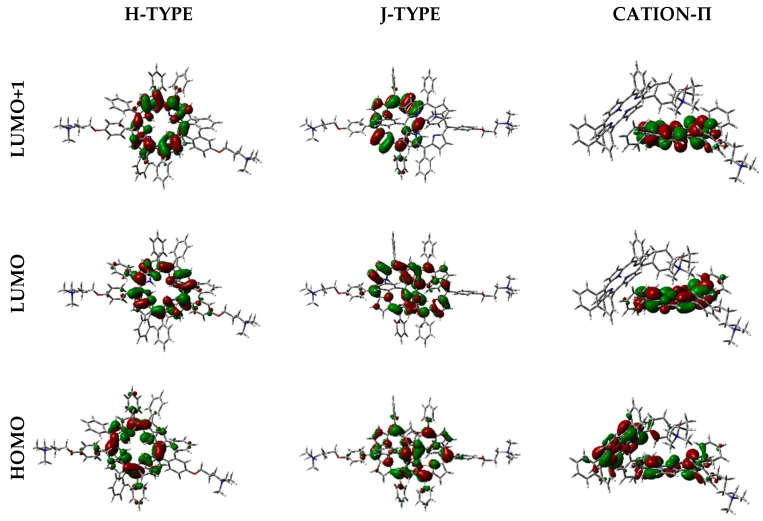

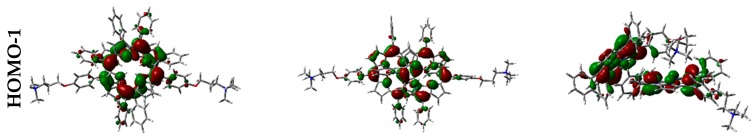
Frontier orbitals representation in the vacuum, calculated with WB97XD functional and 6-31G(d) basis set.

Frontier orbitals for the different dimers have been calculated (Figure 12).

Interestingly, HOMO-1, HOMO, LUMO, and LUMO+1 were delocalized over the porphyrin core, but in different proportions. In some cases, orbitals are located only over one of the two porphyrin components in the dimer, and such distribution indicates that electronic transitions can occur between the two moieties at the supramolecular level.

## 3. Materials and Methods

[5-{4-(3-trimethylammonium)propyloxyphenyl}-10,15,20-triphenylporphyrin] chloride (**1H_2_**) was synthesized as previously reported, and its spectroscopic characterization was fully in agreement with the reported data [36]. UV-vis spectra were measured with a Cary 50 spectrophotometer (Agilent Technologies, Inc., Santa Clara, CA, USA) by using ethanol and ethanol–water mixture as the solvent.

Geometry optimization and frontier orbital calculations were performed with Density Functional Theory approach, using Gaussian 16 rev. A.03 [47]. **1H_2_** structure was optimized first in the vacuum and then in ethanol, using different functionals, namely B3LYP, WB97XD, and CAM-B3LYP, and the 6-31G(d) basis set. Time-dependent DFT (TD-DFT) calculations were performed to study the electronic transitions, using the same functional adopted for geometry optimization and 6-311G(d,p) basis set. Solvent contribution was included using the PCM with ethanol as the solvent. Dimer geometry optimizations was performed in the vacuum with WB97XD and CAM-B3LYP functionals (6-31G(d) basis set), applying correction for BSSE. For each structure, frequency calculations was performed, and no imaginary frequencies were obtained. Considering the optimized geometry of each dimeric structure with WB97XD functional 6-31G(d) basis set and BSSE correction, single-point energy calculations were performed with the same functional and basis set, and using water as the solvent in PCM. Frontier orbitals have been visualized using GaussView 6 (Gaussian Inc., Wallingford, CT, USA).

## 4. Conclusions

Despite a large interest in porphyrin aggregation for several application purposes, DFT studies on porphyrin multimeric forms are still scarce. Therefore, here we proposed a DFT investigation of different plausible dimeric species, using the amphiphilic [5-{4-(3-trimethylammonium)propyloxyphenyl}-10,15,20-triphenylporphyrin] chloride as model substrate. Notably, the experimental spectrum of the aggregated porphyrin in EtOH:H_2_O 1:9 *v*:*v* was quite broad, thus no precise information on the aggregate-type could be deduced.

H-, J-, and cation–π dimers of **1H_2_** were modeled. CAM-B3LYP and WB97XD functionals were screened to define well which aggregate was the most favored in the experimental conditions. Energy values in the vacuum were calculated and compared for all the dimers. Results showed that the cation–π dimer was the most stable with both functionals. Thus, when the solvent is not included in the simulation, the ammonium group interaction with the electron-rich macrocycle stabilizes the structure, which was energetically favored. However, geometry optimization of H- and J- dimers performed with CAM-B3LYP functional led to unsatisfactory results, since interplanar distance between porphyrin planes was too high to allow π–π interactions.

Therefore, single-point energy calculations were performed with WB97XD, using water as the solvent in PCM. Results showed that the H-type aggregate, where the porphyrin cores are stacked and the positive-charged substituents are located at the opposite sides of the molecules, was the most stable structure. It can be anticipated that water solvation of the ammonium groups confers high stability to such a dimer, thus becoming the prevalent structure in aqueous solution. Conversely, the cation–π dimer in water was slightly unfavored with respect to the H-type, likely because water molecules can compete in the ammonium cation interaction with the porphyrin core. However, the energy gap between these two species was only 0.2 kcal·mol^−1^_,_ and therefore the co-presence of both structures in water can be expected.

Moreover, orbital distribution in the monomer and in the different dimeric forms has been calculated. Frontier orbitals were delocalized over the porphyrin core, being responsible for π→π* transitions. The interesting feature about orbital distribution in the case of the dimers was that they can be located over only one of the porphyrin components, indicating that electronic transitions can occur between the two units.

To conclude, WB97XD was an appropriate functional for modeling amphiphilic porphyrin dimers, which can also be adopted for other molecules, as well as for larger supramolecular structures.

## Figures and Tables

**Table 1:** Calculated energy differences among the dimers in the vacuum with CAM-B3LYP and WB97XD functionals and 6-31G(d) basis set, considering the counterpoise correction.

Modeled Dimeric Structure	ΔE_CAM-B3LYP_ (kcal·mol^−1^)	ΔE_WB97XD_ (kcal·mol^−1^)
H	5.7	12.1
J	3.5	16.1
Cation-π	-	-

**Table 2:** Single-point energy differences among the dimers in water (PCM) with WB97XD functional and 6-31G(d) basis set. Geometry optimization performed with WB97XD functional 6-31G(d) basis set, with CP, in the vacuum.

Dimeric Structure	ΔE_WB97XD_ (kcal·mol^−1^)
H	-
J	7.2
Cation-π	0.2

## Supplementary Materials

The supplementary materials are available online.
