# Supplementary material for: Amphiphilic Porphyrin Aggregates: A DFT Investigation
Source: Molecules. 2019 Dec 29;25(1):133. doi: 10.3390/molecules25010133 (PMC6982950; doi:10.3390/molecules25010133)
Supplement: Supplementary file 1 [file molecules-25-00133-s001.pdf]

Supplementary Materials

Amphiphilic Porphyrin Aggregates: a DFT Investigation

Federica Sabuzi *, Manuela Stefanelli, Donato Monti, Valeria Conte and Pierluca Galloni *

Department of Chemical Sciences and Technologies, University of Rome Tor Vergata, Via della Ricerca Scientifica snc, 00133 Rome, Italy; manuela.stefanelli@uniroma2.it (M.S.); monti@stc.uniroma2.it (D.M.); valeria.conte@uniroma2.it (V.C.)

* Correspondence: federica.sabuzi@uniroma2.it (F.S.); galloni@scienze.uniroma2.it (P.G.); Tel.: + 39-06-7259-4490 (F.S.); + 39-06-7259-4380 (P.G.)

Table of contents

Fig. S1. Geometry optimization of **1H$_2$** in EtOH with WB97XD 6-31G(d) 3

Optimized cartesian coordinates (in Angstroms) 4

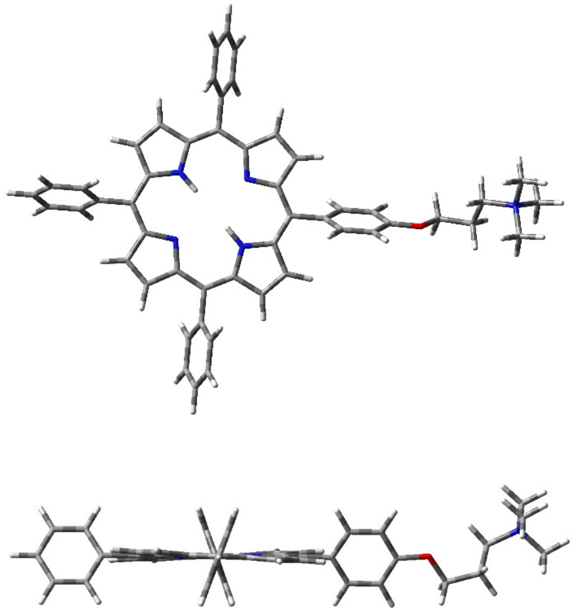

Figure S1. Geometry optimization of **$1H_2$** in EtOH with WB97XD functional and 6-31G(d) basis set, front and side view. C atoms (in grey), H atoms (in white), O atoms (in red) and N atoms (in blue).

Optimized cartesian coordinates (in Angstroms)

- Geometry optimization of **1H₂** in EtOH (WB97XD functional 6-31G(d) basis set)

```
Energy -2279,906671 Hartree
Stoichiometry    C50H44N5O(1+)
 Framework group  C1[X(C50H44N5O)]
 Deg. of freedom   294
 Full point group                 C1      NOp   1
 Largest Abelian subgroup         C1      NOp   1
 Largest concise Abelian subgroup C1      NOp   1
                         Standard orientation:
```

Center Number	Atomic Number	Atomic Type	Coordinates (Angstroms) X	Y	Z
1	7	0	-3.407386	1.594867	0.042531
2	7	0	-0.505155	1.339339	-0.098916
3	7	0	-0.653745	-1.570633	-0.053766
4	7	0	-3.557727	-1.314481	0.032034
5	6	0	-4.768882	1.443244	0.044040
6	6	0	-5.327502	2.758387	-0.050066
7	6	0	-4.299224	3.652778	-0.094817
8	6	0	-3.070090	2.920808	-0.029426
9	6	0	-1.777678	3.452114	-0.037909
10	6	0	-0.592537	2.696189	-0.032904
11	6	0	0.728012	3.302840	0.057556
12	6	0	1.614058	2.285053	0.029264
13	6	0	0.829001	1.063586	-0.080565
14	6	0	1.412007	-0.212659	-0.150883
15	6	0	0.703060	-1.418952	-0.167954
16	6	0	1.251069	-2.733132	-0.309335
17	6	0	0.222150	-3.628165	-0.265958
18	6	0	-0.995800	-2.896308	-0.097678
19	6	0	-2.286312	-3.427773	-0.001716
20	6	0	-3.466142	-2.672512	0.093140
21	6	0	-4.777259	-3.277873	0.281379
22	6	0	-5.662193	-2.259589	0.315428
23	6	0	-4.885792	-1.038895	0.147339
24	6	0	-5.472339	0.237947	0.119557
25	1	0	-6.384863	2.974728	-0.087703
26	1	0	-4.369928	4.727369	-0.175492
27	1	0	0.933481	4.359968	0.148185
28	1	0	2.691354	2.341073	0.093207
29	1	0	2.300910	-2.950256	-0.439197
30	1	0	0.285874	-4.702653	-0.353294
31	1	0	-4.976412	-4.334734	0.387769
32	1	0	-6.732438	-2.314153	0.455136
33	1	0	-1.315565	-0.806076	0.019025
34	1	0	-2.742269	0.829965	0.060576
35	6	0	2.899997	-0.311767	-0.214868
36	6	0	3.604332	0.178872	-1.314041
37	6	0	3.628003	-0.902285	0.826905
38	6	0	4.993261	0.092683	-1.388010
39	1	0	3.060067	0.636167	-2.135168
40	6	0	5.008918	-0.995566	0.770135

41	1	0	3.101848	-1.287506	1.695529
42	6	0	5.699552	-0.497611	-0.339462
43	1	0	5.500591	0.481057	-2.263277
44	1	0	5.572866	-1.447732	1.579496
45	6	0	-2.396566	-4.918110	-0.006874
46	6	0	-3.008859	-5.580083	-1.076072
47	6	0	-1.888369	-5.673792	1.054547
48	6	0	-3.111920	-6.968084	-1.083349
49	1	0	-3.403056	-4.999902	-1.905488
50	6	0	-1.991261	-7.062068	1.047917
51	1	0	-1.414948	-5.166943	1.890693
52	6	0	-2.603313	-7.712425	-0.021181
53	1	0	-3.587662	-7.468269	-1.921484
54	1	0	-1.595889	-7.635263	1.881054
55	1	0	-2.683843	-8.795183	-0.026536
56	6	0	-6.962748	0.335231	0.170190
57	6	0	-7.740709	-0.136625	-0.892072
58	6	0	-7.602498	0.900764	1.277829
59	6	0	-9.128897	-0.046052	-0.847402
60	1	0	-7.250679	-0.574256	-1.757121
61	6	0	-8.990912	0.991812	1.323047
62	1	0	-7.005580	1.265804	2.108839
63	6	0	-9.757310	0.518594	0.260452
64	1	0	-9.719635	-0.414173	-1.680761
65	1	0	-9.473738	1.429899	2.191335
66	1	0	-10.840197	0.589363	0.295508
67	6	0	-1.667135	4.942342	-0.054951
68	6	0	-2.089301	5.700999	1.041482
69	6	0	-1.140134	5.601355	-1.170458
70	6	0	-1.986071	7.089183	1.023564
71	1	0	-2.496009	5.196571	1.913441
72	6	0	-1.036547	6.989199	-1.188987
73	1	0	-0.813354	5.018912	-2.027178
74	6	0	-1.459366	7.736501	-0.091800
75	1	0	-2.314516	7.664638	1.883782
76	1	0	-0.628179	7.487065	-2.063277
77	1	0	-1.378810	8.819179	-0.106114
78	8	0	7.050542	-0.638267	-0.305242
79	6	0	7.814880	-0.117223	-1.380540
80	1	0	7.545623	-0.615999	-2.318787
81	1	0	7.627270	0.958881	-1.493091
82	6	0	9.279933	-0.381792	-1.065321
83	1	0	9.417727	-1.461785	-0.964529
84	1	0	9.868390	-0.041244	-1.921430
85	6	0	9.675181	0.355581	0.207073
86	1	0	9.011794	0.076604	1.027546
87	1	0	9.601920	1.435504	0.057070
88	7	0	11.083636	0.097197	0.698235
89	6	0	12.090930	0.434704	-0.356118
90	1	0	11.996204	-0.265065	-1.184699
91	1	0	11.912660	1.454261	-0.698336
92	1	0	13.086823	0.353964	0.078753
93	6	0	11.317865	0.974556	1.890866
94	1	0	10.569395	0.743269	2.648578
95	1	0	12.317226	0.780259	2.279437
96	1	0	11.232272	2.016478	1.582796
97	6	0	11.252036	-1.331708	1.113208
98	1	0	12.250210	-1.453857	1.532998
99	1	0	10.497671	-1.570583	1.862701

100	1	0	11.140238	-1.978876	0.245402

Rotational constants (GHZ): 0.0572531 0.0218010 0.0162381

- Geometry optimization of **1H₂** in EtOH (CAM-B3LYP functional 6-31G(d) basis set)

```
Energy -2279,361705 Hartree
Stoichiometry    C50H44N5O(1+)
 Framework group  C1[X(C50H44N5O)]
 Deg. of freedom   294
 Full point group                 C1      NOp   1
 Largest Abelian subgroup         C1      NOp   1
 Largest concise Abelian subgroup C1      NOp   1
                        Standard orientation:
```

Center Number	Atomic Number	Atomic Type	Coordinates (Angstroms) X	Y	Z
1	7	0	-3.418505	1.591090	0.043263
2	7	0	-0.512545	1.346546	-0.097454
3	7	0	-0.651362	-1.567210	-0.048456
4	7	0	-3.559531	-1.322350	0.026196
5	6	0	-4.780082	1.433481	0.043409
6	6	0	-5.340958	2.746659	-0.047710
7	6	0	-4.316861	3.644106	-0.089506
8	6	0	-3.084998	2.918949	-0.025390
9	6	0	-1.794699	3.455608	-0.035187
10	6	0	-0.607636	2.702858	-0.032795
11	6	0	0.711184	3.311189	0.056849
12	6	0	1.599575	2.297701	0.029612
13	6	0	0.821570	1.072727	-0.078669
14	6	0	1.410740	-0.201264	-0.143395
15	6	0	0.705959	-1.409672	-0.157233
16	6	0	1.257177	-2.722235	-0.292512
17	6	0	0.232510	-3.620073	-0.251422
18	6	0	-0.989344	-2.894792	-0.090517
19	6	0	-2.277835	-3.431657	-0.001986
20	6	0	-3.460525	-2.679476	0.085535
21	6	0	-4.770598	-3.286864	0.267494
22	6	0	-5.658129	-2.273045	0.299578
23	6	0	-4.888250	-1.048645	0.136723
24	6	0	-5.480127	0.225745	0.112577
25	1	0	-6.398022	2.960069	-0.085387
26	1	0	-4.392500	4.717684	-0.167316
27	1	0	0.913453	4.368033	0.146057
28	1	0	2.675667	2.357881	0.093160
29	1	0	2.307449	-2.936314	-0.416651
30	1	0	0.301374	-4.693644	-0.335323
31	1	0	-4.966993	-4.343485	0.371177
32	1	0	-6.727715	-2.332231	0.434637
33	1	0	-1.316360	-0.804900	0.018459
34	1	0	-2.750361	0.828747	0.058644
35	6	0	2.899913	-0.294133	-0.203332
36	6	0	3.607569	0.198138	-1.298536
37	6	0	3.629064	-0.881145	0.838713

38	6	0	4.995816	0.116483	-1.368744
39	1	0	3.065778	0.651828	-2.122404
40	6	0	5.009081	-0.968525	0.786159
41	1	0	3.103853	-1.267059	1.706744
42	6	0	5.700417	-0.469623	-0.319901
43	1	0	5.505994	0.505739	-2.240910
44	1	0	5.573663	-1.417648	1.596214
45	6	0	-2.383308	-4.922815	-0.006051
46	6	0	-2.993089	-5.589971	-1.072202
47	6	0	-1.872505	-5.677743	1.053439
48	6	0	-3.090229	-6.976890	-1.078892
49	1	0	-3.388607	-5.014307	-1.903249
50	6	0	-1.970621	-7.064857	1.048485
51	1	0	-1.402125	-5.170616	1.890237
52	6	0	-2.579488	-7.718296	-0.018101
53	1	0	-3.563723	-7.478876	-1.916899
54	1	0	-1.573188	-7.635176	1.882290
55	1	0	-2.655886	-8.801071	-0.022626
56	6	0	-6.971467	0.317065	0.161083
57	6	0	-7.748364	-0.159711	-0.898512
58	6	0	-7.616700	0.882571	1.264340
59	6	0	-9.135382	-0.073715	-0.856132
60	1	0	-7.257951	-0.596053	-1.763149
61	6	0	-9.003993	0.967779	1.308345
62	1	0	-7.024393	1.250795	2.096356
63	6	0	-9.767173	0.490143	0.247853
64	1	0	-9.723084	-0.445580	-1.689659
65	1	0	-9.488754	1.405526	2.175423
66	1	0	-10.850070	0.556887	0.281500
67	6	0	-1.688878	4.946674	-0.050211
68	6	0	-2.119108	5.704521	1.042448
69	6	0	-1.158990	5.610923	-1.160021
70	6	0	-2.020631	7.091553	1.027038
71	1	0	-2.526845	5.199771	1.912874
72	6	0	-1.061453	6.997710	-1.177061
73	1	0	-0.826809	5.033032	-2.016874
74	6	0	-1.491788	7.742023	-0.083126
75	1	0	-2.355235	7.664079	1.886511
76	1	0	-0.651231	7.497430	-2.049108
77	1	0	-1.415382	8.824733	-0.095930
78	8	0	7.054759	-0.603966	-0.282879
79	6	0	7.820486	-0.106044	-1.370124
80	1	0	7.535543	-0.610339	-2.299806
81	1	0	7.645506	0.969841	-1.494786
82	6	0	9.282610	-0.388528	-1.062751
83	1	0	9.404421	-1.468799	-0.950673
84	1	0	9.870364	-0.069422	-1.926886
85	6	0	9.700381	0.354337	0.198120
86	1	0	9.046335	0.084939	1.027862
87	1	0	9.632643	1.433187	0.043892
88	7	0	11.114280	0.092654	0.675685
89	6	0	12.113587	0.430252	-0.386613
90	1	0	12.002422	-0.258967	-1.220857
91	1	0	11.943286	1.454709	-0.716080
92	1	0	13.112486	0.335103	0.036820
93	6	0	11.361085	0.968527	1.866912
94	1	0	10.622639	0.734567	2.632658
95	1	0	12.364990	0.775375	2.242372
96	1	0	11.269292	2.010175	1.561912

97	6	0	11.285274	-1.336697	1.088662
98	1	0	12.288747	-1.460773	1.493426
99	1	0	10.542592	-1.572904	1.849797
100	1	0	11.157556	-1.983202	0.223495

Rotational constants (GHZ): 0.0572358 0.0217379 0.0161953

- Geometry optimization of **1H$_2$** in EtOH (B3LYP functional 6-31G+dp basis set)

Energy -2280,780863 Hartree
Stoichiometry C50H44N5O(1+)
Framework group C1[X(C50H44N5O)]
Deg. of freedom 294
Full point group C1 NOp 1
Largest Abelian subgroup C1 NOp 1
Largest concise Abelian subgroup C1 NOp 1

Standard orientation:

Center Number	Atomic Number	Atomic Type	Coordinates (Angstroms) X	Y	Z
1	7	0	-3.411566	1.630525	0.035185
2	7	0	-0.490024	1.332757	-0.091708
3	7	0	-0.687619	-1.600528	-0.031890
4	7	0	-3.611230	-1.302557	0.022854
5	6	0	-4.783424	1.496532	0.029531
6	6	0	-5.320845	2.824010	-0.062363
7	6	0	-4.273335	3.708598	-0.100436
8	6	0	-3.051287	2.959265	-0.033628
9	6	0	-1.743320	3.474154	-0.041154
10	6	0	-0.560930	2.699960	-0.034626
11	6	0	0.773470	3.283662	0.048957
12	6	0	1.648726	2.245465	0.028404
13	6	0	0.846498	1.030820	-0.068501
14	6	0	1.410325	-0.264220	-0.120620
15	6	0	0.680861	-1.466471	-0.129257
16	6	0	1.211468	-2.794284	-0.246865
17	6	0	0.163945	-3.679326	-0.210598
18	6	0	-1.051257	-2.929533	-0.069322
19	6	0	-2.357487	-3.444110	0.008888
20	6	0	-3.536784	-2.669533	0.085638
21	6	0	-4.863516	-3.252050	0.256389
22	6	0	-5.738537	-2.213972	0.281204
23	6	0	-4.943781	-1.000418	0.124958
24	6	0	-5.509236	0.294417	0.095911
25	1	0	-6.373427	3.059458	-0.102641
26	1	0	-4.331702	4.783645	-0.177066
27	1	0	0.999709	4.337045	0.128338
28	1	0	2.726466	2.288635	0.088248
29	1	0	2.259253	-3.029568	-0.355607
30	1	0	0.217443	-4.754806	-0.284587
31	1	0	-5.084565	-4.304606	0.357864
32	1	0	-6.810666	-2.256862	0.406634
33	1	0	-1.340084	-0.826483	0.023213
34	1	0	-2.756929	0.856557	0.054198
35	6	0	2.903756	-0.385114	-0.168990

36	6	0	3.622276	-0.010112	-1.311087
37	6	0	3.627468	-0.877734	0.932513
38	6	0	5.016576	-0.116735	-1.369793
39	1	0	3.089199	0.366753	-2.179103
40	6	0	5.014517	-0.982036	0.895149
41	1	0	3.097664	-1.172937	1.833536
42	6	0	5.717411	-0.600773	-0.258116
43	1	0	5.530561	0.177035	-2.276892
44	1	0	5.567960	-1.355157	1.751148
45	6	0	-2.490870	-4.938543	0.007497
46	6	0	-3.035025	-5.606286	-1.102107
47	6	0	-2.073729	-5.697997	1.112992
48	6	0	-3.158291	-6.998562	-1.106893
49	1	0	-3.356387	-5.031755	-1.966087
50	6	0	-2.201620	-7.090043	1.111407
51	1	0	-1.656534	-5.194490	1.980409
52	6	0	-2.743240	-7.744718	0.000722
53	1	0	-3.577357	-7.498282	-1.975490
54	1	0	-1.879700	-7.660581	1.977810
55	1	0	-2.841232	-8.826328	-0.001659
56	6	0	-7.004248	0.415305	0.137687
57	6	0	-7.784305	0.025325	-0.963441
58	6	0	-7.651667	0.921627	1.276693
59	6	0	-9.176916	0.139614	-0.927264
60	1	0	-7.296295	-0.362581	-1.852940
61	6	0	-9.044701	1.031356	1.315899
62	1	0	-7.061360	1.221742	2.137768
63	6	0	-9.811546	0.641796	0.213304
64	1	0	-9.763794	-0.162588	-1.789841
65	1	0	-9.528647	1.419443	2.207512
66	1	0	-10.893705	0.728732	0.242580
67	6	0	-1.610904	4.968678	-0.055911
68	6	0	-1.953062	5.730452	1.073495
69	6	0	-1.142785	5.634061	-1.200966
70	6	0	-1.826197	7.122519	1.060372
71	1	0	-2.311254	5.228820	1.967956
72	6	0	-1.020668	7.026405	-1.216975
73	1	0	-0.880344	5.057718	-2.083459
74	6	0	-1.360627	7.774891	-0.085575
75	1	0	-2.089943	7.694832	1.945070
76	1	0	-0.661573	7.524432	-2.112983
77	1	0	-1.263935	8.856563	-0.097022
78	8	0	7.078635	-0.738465	-0.199737
79	6	0	7.862099	-0.386096	-1.346538
80	1	0	7.585579	-1.018777	-2.197614
81	1	0	7.680159	0.661913	-1.616752
82	6	0	9.330184	-0.613404	-0.995243
83	1	0	9.453123	-1.656944	-0.691558
84	1	0	9.909128	-0.457955	-1.909985
85	6	0	9.770733	0.351346	0.104922
86	1	0	9.134177	0.245193	0.984617
87	1	0	9.703831	1.383069	-0.247901
88	7	0	11.204062	0.178110	0.605867
89	6	0	12.195532	0.313681	-0.521423
90	1	0	12.066021	-0.512037	-1.217977
91	1	0	12.027896	1.265421	-1.025258
92	1	0	13.200124	0.283930	-0.100832
93	6	0	11.468813	1.264784	1.619967
94	1	0	10.750298	1.168066	2.433247

95	1	0	12.483099	1.148036	1.999494
96	1	0	11.359593	2.233643	1.133973
97	6	0	11.386534	-1.159664	1.277520
98	1	0	12.394668	-1.201921	1.688376
99	1	0	10.651555	-1.253779	2.076366
100	1	0	11.255174	-1.954636	0.546699

Rotational constants (GHZ): 0.0566080 0.0214573 0.0159991

- Geometry optimization of H-dimer in the *vacuum* (CAM-B3LYP functional 6-31G(d) basis set) with BSSE correction.

```
Energy -4558,533314 Hartree
Stoichiometry    C100H88N10O2(2+)
 Framework group  C1[X(C100H88N10O2)]
 Deg. of freedom   594
 Full point group                 C1      NOp   1
 Largest Abelian subgroup         C1      NOp   1
 Largest concise Abelian subgroup C1      NOp   1
                        Standard orientation:
```

Center Number	Atomic Number	Atomic Type	Coordinates (Angstroms) X	Y	Z
1	7	0	4.099424	0.497825	1.760507
2	7	0	3.531439	-2.308974	1.198891
3	7	0	0.770888	-2.031920	2.110629
4	7	0	1.320406	0.790050	2.591424
5	6	0	4.089555	1.837055	2.051487
6	6	0	5.379791	2.339196	1.693102
7	6	0	6.119938	1.301714	1.211703
8	6	0	5.314540	0.119742	1.256208
9	6	0	5.686384	-1.167120	0.856319
10	6	0	4.847726	-2.294383	0.863235
11	6	0	5.312817	-3.633699	0.528518
12	6	0	4.248623	-4.449337	0.663727
13	6	0	3.137301	-3.606189	1.078563
14	6	0	1.851031	-4.110744	1.319646
15	6	0	0.764922	-3.359003	1.776139
16	6	0	-0.575109	-3.821830	1.965925
17	6	0	-1.326938	-2.773489	2.403488
18	6	0	-0.476328	-1.628502	2.505180
19	6	0	-0.842159	-0.346960	2.924195
20	6	0	0.020125	0.758063	2.988863
21	6	0	-0.390110	2.043928	3.536622
22	6	0	0.684333	2.849940	3.453402
23	6	0	1.744510	2.055820	2.845962
24	6	0	3.023927	2.571104	2.579575
25	1	0	5.680015	3.371351	1.787118
26	1	0	7.132414	1.340821	0.840336
27	1	0	6.321811	-3.904400	0.254705
28	1	0	4.206832	-5.518636	0.519150
29	1	0	-0.903885	-4.831234	1.772155
30	1	0	-2.381563	-2.770724	2.631457
31	1	0	-1.363670	2.273824	3.942538
32	1	0	0.771867	3.875513	3.778474

33	1	0	1.578198	-1.421503	2.047296
34	1	0	3.303657	-0.120808	1.873948
35	6	0	1.599079	-5.563415	1.068344
36	6	0	1.569204	-6.064831	-0.235166
37	6	0	1.385870	-6.443086	2.132130
38	6	0	1.335380	-7.415213	-0.468122
39	1	0	1.721514	-5.385186	-1.067990
40	6	0	1.153674	-7.793856	1.899463
41	1	0	1.411588	-6.062299	3.148597
42	6	0	1.128359	-8.283495	0.598268
43	1	0	1.311682	-7.789195	-1.487194
44	1	0	0.996878	-8.465596	2.737854
45	1	0	0.948154	-9.338415	0.416029
46	6	0	-2.266924	-0.153406	3.335101
47	6	0	-3.129974	0.616006	2.551324
48	6	0	-2.756599	-0.727253	4.510895
49	6	0	-4.450185	0.814927	2.939421
50	1	0	-2.762830	1.056418	1.629804
51	6	0	-4.076497	-0.529425	4.899546
52	1	0	-2.090271	-1.320433	5.129736
53	6	0	-4.925652	0.244538	4.115690
54	1	0	-5.104892	1.419550	2.319090
55	1	0	-4.437592	-0.971847	5.823035
56	1	0	-5.953416	0.409136	4.425809
57	6	0	3.283103	4.014719	2.866102
58	6	0	2.619113	5.013510	2.149161
59	6	0	4.194709	4.394354	3.854677
60	6	0	2.861520	6.356519	2.411352
61	1	0	1.906324	4.730099	1.381167
62	6	0	4.436383	5.737404	4.119337
63	1	0	4.707024	3.626819	4.426688
64	6	0	3.771387	6.722275	3.397440
65	1	0	2.337834	7.118163	1.842060
66	1	0	5.141203	6.014467	4.897229
67	1	0	3.958640	7.771110	3.605087
68	6	0	7.098881	-1.340135	0.400980
69	6	0	8.161950	-1.181405	1.294928
70	6	0	7.391805	-1.669707	-0.925363
71	6	0	9.477945	-1.343386	0.880482
72	1	0	7.949754	-0.939534	2.331117
73	6	0	8.704205	-1.833962	-1.351405
74	1	0	6.577222	-1.796763	-1.630634
75	6	0	9.740049	-1.669323	-0.443024
76	1	0	10.298830	-1.237425	1.583017
77	1	0	8.930443	-2.095650	-2.379957
78	7	0	-0.817073	2.131669	-2.296269
79	7	0	-2.946091	0.193210	-1.796781
80	7	0	-4.756917	2.274772	-0.867319
81	7	0	-2.654896	4.219185	-1.439269
82	6	0	-0.032236	3.226940	-2.539610
83	6	0	1.182857	2.737765	-3.115146
84	6	0	1.101523	1.379974	-3.184440
85	6	0	-0.171591	0.983595	-2.666641
86	6	0	-0.668714	-0.320733	-2.587974
87	6	0	-1.962247	-0.668052	-2.172479
88	6	0	-2.422891	-2.047609	-2.096398
89	6	0	-3.705455	-1.996031	-1.688767
90	6	0	-4.020517	-0.585395	-1.507014
91	6	0	-5.272049	-0.133956	-1.059946

92	6	0	-5.595805	1.196102	-0.777647
93	6	0	-6.859480	1.701294	-0.337201
94	6	0	-6.744441	3.049318	-0.170568
95	6	0	-5.407923	3.427305	-0.509632
96	6	0	-4.879143	4.720456	-0.502521
97	6	0	-3.584881	5.065999	-0.923409
98	6	0	-3.069041	6.426017	-0.850415
99	6	0	-1.820367	6.383275	-1.351964
100	6	0	-1.575492	4.995907	-1.722869
101	6	0	-0.358574	4.559564	-2.272232
102	1	0	2.001185	3.362930	-3.437064
103	1	0	1.841306	0.696667	-3.571949
104	1	0	-1.828166	-2.921711	-2.315542
105	1	0	-4.375473	-2.822541	-1.502873
106	1	0	-7.740350	1.097494	-0.181972
107	1	0	-7.511629	3.740473	0.143092
108	1	0	-3.597284	7.279988	-0.453874
109	1	0	-1.115806	7.195410	-1.449415
110	1	0	-3.804366	2.234919	-1.214332
111	1	0	-1.760597	2.172968	-1.927163
112	6	0	-6.360398	-1.139277	-0.866143
113	6	0	-6.942826	-1.782705	-1.964477
114	6	0	-6.834316	-1.457638	0.404354
115	6	0	-7.966492	-2.700035	-1.798925
116	1	0	-6.586359	-1.551153	-2.962834
117	6	0	-7.865265	-2.377438	0.589338
118	1	0	-6.384469	-0.984622	1.271572
119	6	0	-8.432069	-2.992479	-0.519793
120	1	0	-8.419258	-3.195641	-2.651180
121	1	0	-8.199486	-2.600841	1.595643
122	6	0	-5.784182	5.808785	-0.021011
123	6	0	-6.266509	6.776835	-0.905351
124	6	0	-6.166262	5.876905	1.320906
125	6	0	-7.108904	7.788049	-0.458869
126	1	0	-5.977834	6.730371	-1.950805
127	6	0	-7.007594	6.888902	1.768737
128	1	0	-5.788810	5.135067	2.018177
129	6	0	-7.481557	7.847036	0.879646
130	1	0	-7.476127	8.531278	-1.159760
131	1	0	-7.287741	6.932297	2.816756
132	1	0	-8.137077	8.638587	1.228889
133	6	0	0.678234	5.584956	-2.598859
134	6	0	0.447062	6.531697	-3.600373
135	6	0	1.895101	5.620145	-1.913348
136	6	0	1.406571	7.488859	-3.907430
137	1	0	-0.493008	6.510167	-4.142633
138	6	0	2.854679	6.578510	-2.218216
139	1	0	2.084370	4.893353	-1.129536
140	6	0	2.613138	7.515609	-3.216530
141	1	0	1.211039	8.214514	-4.690778
142	1	0	3.791117	6.595220	-1.669005
143	1	0	3.361462	8.264972	-3.454897
144	6	0	0.267946	-1.412220	-2.999018
145	6	0	1.371285	-1.735560	-2.205714
146	6	0	0.063057	-2.119319	-4.186440
147	6	0	2.250107	-2.741985	-2.591079
148	1	0	1.536987	-1.202490	-1.274726
149	6	0	0.941307	-3.124764	-4.573091
150	1	0	-0.789188	-1.870174	-4.811204

151	6	0	2.037876	-3.437678	-3.776470
152	1	0	3.096633	-2.983237	-1.955513
153	1	0	0.770865	-3.660848	-5.501806
154	1	0	2.724850	-4.222025	-4.080252
155	8	0	11.051714	-1.865769	-0.861946
156	8	0	-9.462531	-3.906063	-0.453947
157	6	0	-9.999158	-4.221935	0.808286
158	1	0	-10.377097	-3.319592	1.309521
159	1	0	-9.242667	-4.675852	1.459492
160	6	0	-11.127108	-5.220420	0.565636
161	1	0	-11.551934	-5.507224	1.531877
162	1	0	-10.688771	-6.110142	0.105339
163	6	0	-12.168851	-4.583430	-0.343349
164	1	0	-11.678879	-4.185437	-1.233006
165	1	0	-12.672625	-3.759845	0.169512
166	6	0	11.732396	-0.683592	-1.237443
167	1	0	11.205894	-0.171429	-2.051555
168	1	0	11.795233	0.013674	-0.389973
169	6	0	13.122331	-1.101148	-1.707485
170	1	0	13.672344	-0.208055	-2.017827
171	1	0	12.993843	-1.746850	-2.580591
172	6	0	13.828052	-1.836715	-0.577242
173	1	0	13.172205	-2.617578	-0.189629
174	1	0	14.065658	-1.148757	0.238495
175	7	0	-13.275675	-5.500366	-0.837973
176	7	0	15.138688	-2.517052	-0.938491
177	6	0	-12.720610	-6.587652	-1.707255
178	1	0	-13.549709	-7.169367	-2.110652
179	1	0	-12.152262	-6.134323	-2.519602
180	1	0	-12.074339	-7.233545	-1.116587
181	6	0	-14.225704	-4.674655	-1.653224
182	1	0	-13.679985	-4.223491	-2.481965
183	1	0	-15.018802	-5.317793	-2.035089
184	1	0	-14.651393	-3.895253	-1.021177
185	6	0	-14.022201	-6.108442	0.308420
186	1	0	-13.355602	-6.760795	0.869611
187	1	0	-14.394260	-5.310444	0.951771
188	1	0	-14.858080	-6.687946	-0.084263
189	6	0	16.118071	-1.533013	-1.498244
190	1	0	15.739974	-1.142364	-2.441299
191	1	0	16.251236	-0.720425	-0.783329
192	1	0	17.069065	-2.039450	-1.665227
193	6	0	15.708412	-3.111639	0.314435
194	1	0	14.989404	-3.818839	0.728070
195	1	0	16.639788	-3.624557	0.073735
196	1	0	15.898798	-2.313291	1.031758
197	6	0	14.912806	-3.618467	-1.929434
198	1	0	15.857461	-4.136188	-2.097160
199	1	0	14.173599	-4.310308	-1.525250
200	1	0	14.554786	-3.198508	-2.867031

Rotational constants (GHZ): 0.0168252 0.0071325 0.0057833

- Geometry optimization of J-dimer in the *vacuum* (CAM-B3LYP functional 6-31G(d) basis set) with BSSE correction.

Energy -4558,536836 Hartree
Stoichiometry   C100H88N10O2(2+)
Framework group C1[X(C100H88N10O2)]
Deg. of freedom   594
Full point group   C1   NOp   1
Largest Abelian subgroup   C1   NOp   1
Largest concise Abelian subgroup C1   NOp   1

Standard orientation:

Center Number	Atomic Number	Atomic Type	Coordinates (Angstroms) X	Y	Z
1	7	0	-6.167915	1.985721	-0.795682
2	7	0	-5.502333	-0.840008	-1.086605
3	7	0	-2.757110	-0.210345	-1.859805
4	7	0	-3.422218	2.611602	-1.558742
5	6	0	-6.214256	3.352918	-0.706920
6	6	0	-7.552242	3.689688	-0.328027
7	6	0	-8.257888	2.533111	-0.191858
8	6	0	-7.386596	1.438795	-0.498263
9	6	0	-7.709869	0.079655	-0.495689
10	6	0	-6.821812	-0.968411	-0.793286
11	6	0	-7.238438	-2.362213	-0.870553
12	6	0	-6.142732	-3.067149	-1.211905
13	6	0	-5.060304	-2.102565	-1.343515
14	6	0	-3.761462	-2.467840	-1.724382
15	6	0	-2.707740	-1.575343	-1.953020
16	6	0	-1.366808	-1.910598	-2.318559
17	6	0	-0.659869	-0.751973	-2.446674
18	6	0	-1.535786	0.337654	-2.149856
19	6	0	-1.210679	1.697883	-2.153360
20	6	0	-2.101659	2.740629	-1.862039
21	6	0	-1.689637	4.136819	-1.812039
22	6	0	-2.786313	4.842691	-1.479068
23	6	0	-3.863808	3.874090	-1.325480
24	6	0	-5.165836	4.242443	-0.945372
25	1	0	-7.908586	4.697644	-0.181361
26	1	0	-9.292835	2.431488	0.096283
27	1	0	-8.239129	-2.734646	-0.709044
28	1	0	-6.062520	-4.130026	-1.383858
29	1	0	-1.005582	-2.917439	-2.460085
30	1	0	0.379001	-0.643797	-2.717497
31	1	0	-0.690345	4.503734	-1.991398
32	1	0	-2.871726	5.908332	-1.328424
33	1	0	-3.583598	0.325579	-1.618275
34	1	0	-5.343158	1.453684	-1.051735
35	6	0	-3.460899	-3.920349	-1.913182
36	6	0	-3.438605	-4.789209	-0.819523
37	6	0	-3.197573	-4.436030	-3.184520
38	6	0	-3.160852	-6.140092	-0.992387
39	1	0	-3.636842	-4.396461	0.173019
40	6	0	-2.920426	-5.787030	-3.358474
41	1	0	-3.219290	-3.770355	-4.041917
42	6	0	-2.901168	-6.642767	-2.262641
43	1	0	-3.144123	-6.800878	-0.131083
44	1	0	-2.723659	-6.172150	-4.354262
45	1	0	-2.685174	-7.697923	-2.398235
46	6	0	0.205080	2.044935	-2.488313
47	6	0	1.240950	1.743397	-1.601240

48	6	0	0.514375	2.674892	-3.696175
49	6	0	2.557303	2.062728	-1.915084
50	1	0	1.014012	1.265681	-0.653546
51	6	0	1.829922	2.994718	-4.010401
52	1	0	-0.284821	2.910089	-4.392381
53	6	0	2.854425	2.688514	-3.120842
54	1	0	3.345918	1.821879	-1.209375
55	1	0	2.054300	3.481121	-4.954807
56	1	0	3.882146	2.937428	-3.367237
57	6	0	-5.468412	5.696865	-0.773523
58	6	0	-5.709510	6.231948	0.494003
59	6	0	-5.514814	6.547201	-1.880809
60	6	0	-5.990323	7.584434	0.651018
61	1	0	-5.665321	5.580899	1.361827
62	6	0	-5.796174	7.899301	-1.724765
63	1	0	-5.330236	6.139785	-2.870027
64	6	0	-6.034957	8.421577	-0.458219
65	1	0	-6.168478	7.985713	1.643959
66	1	0	-5.830550	8.546015	-2.596052
67	1	0	-6.252814	9.477935	-0.336036
68	6	0	-9.125109	-0.268993	-0.165831
69	6	0	-10.167852	0.079323	-1.029114
70	6	0	-9.438855	-0.958883	1.008545
71	6	0	-11.485116	-0.246002	-0.730333
72	1	0	-9.938204	0.602003	-1.951802
73	6	0	-10.752275	-1.291026	1.317105
74	1	0	-8.639380	-1.238735	1.686515
75	6	0	-11.768013	-0.930926	0.443075
76	1	0	-12.289908	0.011113	-1.412093
77	1	0	-10.993758	-1.834146	2.225077
78	7	0	2.928846	-2.026387	1.508503
79	7	0	5.787049	-1.996604	0.915336
80	7	0	5.931990	0.892825	1.284021
81	7	0	3.078773	0.860421	1.886358
82	6	0	1.636752	-1.784049	1.892648
83	6	0	0.981260	-3.053939	1.946928
84	6	0	1.886769	-4.012499	1.605485
85	6	0	3.133805	-3.367568	1.329428
86	6	0	4.339062	-3.991562	0.993146
87	6	0	5.564607	-3.334668	0.809043
88	6	0	6.791233	-4.038597	0.461468
89	6	0	7.753825	-3.100888	0.375233
90	6	0	7.108021	-1.824042	0.651186
91	6	0	7.794767	-0.598957	0.638236
92	6	0	7.226203	0.648672	0.910902
93	6	0	7.867825	1.923837	0.813559
94	6	0	6.953604	2.884973	1.124457
95	6	0	5.714586	2.238927	1.428992
96	6	0	4.513204	2.860882	1.777050
97	6	0	3.296878	2.196623	2.005723
98	6	0	2.084954	2.888602	2.421681
99	6	0	1.132555	1.944162	2.541034
100	6	0	1.768941	0.677573	2.207610
101	6	0	1.078624	-0.544186	2.213572
102	1	0	-0.050641	-3.194512	2.229603
103	1	0	1.728702	-5.079051	1.560981
104	1	0	6.884623	-5.103627	0.310979
105	1	0	8.800213	-3.247149	0.152628
106	1	0	8.895687	2.071486	0.519481

107	1	0	7.101189	3.953949	1.133086
108	1	0	1.994206	3.949887	2.597399
109	1	0	0.098528	2.073589	2.823160
110	1	0	5.225816	0.176761	1.417513
111	1	0	3.643025	-1.313666	1.405662
112	6	0	9.249849	-0.605606	0.299008
113	6	0	10.213167	-0.277865	1.260455
114	6	0	9.692880	-0.939069	-0.978879
115	6	0	11.564229	-0.289026	0.957169
116	1	0	9.892277	-0.020578	2.264609
117	6	0	11.048158	-0.957156	-1.301928
118	1	0	8.964066	-1.191373	-1.742104
119	6	0	11.981674	-0.631944	-0.325836
120	1	0	12.309552	-0.047053	1.707396
121	1	0	11.347508	-1.221581	-2.309189
122	6	0	4.542275	4.350671	1.899097
123	6	0	5.247138	4.969253	2.934296
124	6	0	3.865695	5.151252	0.975209
125	6	0	5.273876	6.354745	3.045112
126	1	0	5.768128	4.354840	3.662282
127	6	0	3.893254	6.536486	1.084965
128	1	0	3.317770	4.679243	0.165295
129	6	0	4.597223	7.142075	2.120212
130	1	0	5.819779	6.819440	3.860454
131	1	0	3.363824	7.144295	0.357712
132	1	0	4.616429	8.223944	2.207146
133	6	0	-0.365502	-0.541077	2.605891
134	6	0	-0.737993	-0.331695	3.935651
135	6	0	-1.363258	-0.743152	1.650043
136	6	0	-2.079005	-0.321990	4.300412
137	1	0	0.032273	-0.177148	4.685027
138	6	0	-2.705786	-0.728396	2.012674
139	1	0	-1.083699	-0.902522	0.613363
140	6	0	-3.065028	-0.518315	3.339396
141	1	0	-2.352827	-0.160160	5.338544
142	1	0	-3.469790	-0.869529	1.254182
143	1	0	-4.112780	-0.504533	3.623680
144	6	0	4.298360	-5.479169	0.842352
145	6	0	3.643433	-6.066220	-0.242565
146	6	0	4.907282	-6.308619	1.787077
147	6	0	3.601137	-7.448746	-0.382059
148	1	0	3.169819	-5.429634	-0.983717
149	6	0	4.865201	-7.691010	1.648707
150	1	0	5.411912	-5.861331	2.637923
151	6	0	4.212243	-8.264878	0.563268
152	1	0	3.091683	-7.888639	-1.233917
153	1	0	5.340034	-8.321380	2.394231
154	1	0	4.179007	-9.344495	0.455279
155	8	0	-13.079735	-1.291226	0.734263
156	8	0	13.344253	-0.621816	-0.534122
157	6	0	13.832342	-1.007286	-1.796990
158	1	0	13.477395	-0.322537	-2.580299
159	1	0	13.499442	-2.018585	-2.058609
160	6	0	15.354961	-0.983469	-1.702569
161	1	0	15.768435	-1.293494	-2.666557
162	1	0	15.650589	-1.718984	-0.949475
163	6	0	15.811159	0.416318	-1.316442
164	1	0	15.265630	0.744536	-0.430658
165	1	0	15.609897	1.122613	-2.126223

166	6	0	-13.820862	-0.304146	1.425411
167	1	0	-13.337706	-0.045331	2.375055
168	1	0	-13.894362	0.615101	0.827028
169	6	0	-15.201407	-0.894476	1.695695
170	1	0	-15.799480	-0.158933	2.241332
171	1	0	-15.064377	-1.770081	2.336262
172	6	0	-15.844431	-1.276486	0.370120
173	1	0	-15.141001	-1.871903	-0.213616
174	1	0	-16.099899	-0.381959	-0.204070
175	7	0	17.288133	0.570449	-0.990985
176	7	0	-17.125347	-2.090372	0.458692
177	6	0	17.653159	-0.224957	0.225765
178	1	0	18.694310	-0.020974	0.476599
179	1	0	17.004917	0.073391	1.049893
180	1	0	17.528145	-1.285958	0.020008
181	6	0	17.547221	2.020291	-0.709488
182	1	0	16.918248	2.336252	0.122820
183	1	0	18.598659	2.150723	-0.452731
184	1	0	17.306750	2.603241	-1.598592
185	6	0	18.140432	0.140171	-2.144126
186	1	0	18.007915	-0.926719	-2.314563
187	1	0	17.844600	0.700656	-3.031526
188	1	0	19.184136	0.346819	-1.906025
189	6	0	-18.164570	-1.370262	1.259900
190	1	0	-17.825211	-1.274079	2.289704
191	1	0	-18.325240	-0.383071	0.825411
192	1	0	-19.091421	-1.943707	1.230365
193	6	0	-17.638770	-2.292444	-0.935314
194	1	0	-16.876133	-2.805500	-1.521286
195	1	0	-18.546961	-2.894273	-0.896668
196	1	0	-17.855893	-1.320430	-1.378255
197	6	0	-16.862789	-3.433520	1.069637
198	1	0	-17.782691	-4.017674	1.036747
199	1	0	-16.080890	-3.934029	0.498199
200	1	0	-16.545563	-3.309246	2.102849

```
---------------------------------------------------------------------
 Rotational constants (GHZ):           0.0228387           0.0048123
0.0042690
```

- Geometry optimization of cation-π dimer in the *vacuum* (CAM-B3LYP functional 6-31G(d) basis set) with BSSE correction.

```
Energy -4558,542412 Hartree
Stoichiometry    C100H88N10O2(2+)
 Framework group  C1[X(C100H88N10O2)]
 Deg. of freedom   594
 Full point group                C1      NOp   1
 Largest Abelian subgroup        C1      NOp   1
 Largest concise Abelian subgroup C1      NOp   1
                        Standard orientation:
```

Center Number	Atomic Number	Atomic Type	Coordinates (Angstroms) X	Y	Z
1	7	0	7.041608	1.754764	0.112952
2	7	0	4.918359	0.428390	1.614958

3	7	0	6.247576	-2.164170	1.383028
4	7	0	8.381722	-0.828914	-0.077775
5	6	0	8.181562	2.142470	-0.538859
6	6	0	8.103020	3.560991	-0.693466
7	6	0	6.934802	3.983692	-0.132614
8	6	0	6.252518	2.840565	0.388296
9	6	0	5.033193	2.833956	1.072357
10	6	0	4.427233	1.699077	1.631278
11	6	0	3.139460	1.743446	2.301882
12	6	0	2.872494	0.478386	2.690618
13	6	0	3.998278	-0.334972	2.254879
14	6	0	4.069168	-1.727355	2.458924
15	6	0	5.117842	-2.551587	2.052232
16	6	0	5.232005	-3.965233	2.258966
17	6	0	6.402866	-4.381741	1.704635
18	6	0	7.062030	-3.240186	1.145175
19	6	0	8.281915	-3.230949	0.468457
20	6	0	8.881737	-2.091021	-0.094625
21	6	0	10.171620	-2.135949	-0.772504
22	6	0	10.432319	-0.877733	-1.170613
23	6	0	9.308949	-0.065386	-0.720216
24	6	0	9.236224	1.315760	-0.943540
25	1	0	8.863484	4.166558	-1.161916
26	1	0	6.572801	4.997788	-0.061125
27	1	0	2.540306	2.629414	2.452400
28	1	0	2.018954	0.127465	3.251884
29	1	0	4.504091	-4.571625	2.776266
30	1	0	6.795904	-5.386333	1.676722
31	1	0	10.779156	-3.017354	-0.911288
32	1	0	11.293032	-0.515699	-1.712226
33	1	0	6.461765	-1.211890	1.107604
34	1	0	6.845279	0.804308	0.407502
35	6	0	2.905146	-2.388815	3.120659
36	6	0	2.583521	-2.136486	4.457531
37	6	0	2.077726	-3.253847	2.397759
38	6	0	1.441736	-2.675220	5.039773
39	1	0	3.237329	-1.501787	5.046881
40	6	0	0.933117	-3.798501	2.968069
41	1	0	2.329478	-3.486631	1.367645
42	6	0	0.596950	-3.478102	4.279014
43	1	0	1.209455	-2.475151	6.080993
44	1	0	0.284093	-4.463367	2.407099
45	6	0	8.987078	-4.543784	0.340673
46	6	0	9.564849	-5.151981	1.457309
47	6	0	9.077916	-5.184581	-0.897113
48	6	0	10.219866	-6.372494	1.338899
49	1	0	9.507499	-4.656882	2.421947
50	6	0	9.732445	-6.404874	-1.015770
51	1	0	8.630226	-4.719832	-1.770301
52	6	0	10.305002	-7.001902	0.102128
53	1	0	10.669977	-6.829060	2.214818
54	1	0	9.794292	-6.890815	-1.984492
55	1	0	10.818244	-7.953697	0.009081
56	6	0	10.371784	1.984890	-1.650652
57	6	0	11.610220	2.133745	-1.022218
58	6	0	10.211447	2.477123	-2.947611
59	6	0	12.664544	2.759251	-1.677017
60	1	0	11.742349	1.758576	-0.012098
61	6	0	11.266238	3.101364	-3.603427

62	1	0	9.254223	2.359798	-3.446667
63	6	0	12.495261	3.244209	-2.969275
64	1	0	13.620222	2.869914	-1.174454
65	1	0	11.128326	3.472948	-4.614021
66	1	0	13.319220	3.731405	-3.480790
67	6	0	4.339979	4.151745	1.210573
68	6	0	3.797559	4.789366	0.091403
69	6	0	4.232054	4.775176	2.455999
70	6	0	3.164025	6.020903	0.215955
71	1	0	3.877113	4.311857	-0.880578
72	6	0	3.597018	6.005647	2.580896
73	1	0	4.662182	4.293498	3.328687
74	6	0	3.061535	6.632366	1.460996
75	1	0	2.755628	6.508021	-0.664695
76	1	0	3.531945	6.481276	3.554611
77	1	0	2.574694	7.597893	1.557040
78	8	0	-0.581183	-3.979347	4.787236
79	6	0	-1.558000	-3.032589	5.182387
80	1	0	-2.316471	-3.614517	5.708042
81	1	0	-1.142296	-2.308667	5.895158
82	6	0	-2.215673	-2.313245	3.991663
83	1	0	-2.328527	-3.056717	3.197739
84	1	0	-3.219410	-1.997691	4.293494
85	6	0	-1.407934	-1.103233	3.526271
86	1	0	-0.353213	-1.352539	3.419597
87	1	0	-1.491215	-0.293086	4.255815
88	7	0	-1.817609	-0.507793	2.194012
89	6	0	-3.257090	-0.100229	2.195998
90	1	0	-3.877645	-0.986884	2.316284
91	1	0	-3.421623	0.586004	3.028282
92	1	0	-3.501903	0.393891	1.252089
93	6	0	-0.971816	0.707320	1.952288
94	1	0	0.078714	0.415212	1.923370
95	1	0	-1.263621	1.150624	1.001286
96	1	0	-1.138253	1.417344	2.762943
97	6	0	-1.571477	-1.478950	1.077186
98	1	0	-1.724614	-0.971217	0.121733
99	1	0	-0.544574	-1.838040	1.155357
100	1	0	-2.264910	-2.313062	1.167385
101	7	0	-2.005595	2.336856	-1.063965
102	7	0	-4.763654	1.634436	-0.404463
103	7	0	-4.446263	-1.046407	-1.512095
104	7	0	-1.677395	-0.364278	-2.117423
105	6	0	-0.674685	2.384305	-1.402976
106	6	0	-0.164879	3.608137	-0.872747
107	6	0	-1.192179	4.268936	-0.263843
108	6	0	-2.375886	3.481134	-0.401533
109	6	0	-3.665888	3.805978	0.036015
110	6	0	-4.778293	2.952193	-0.048867
111	6	0	-6.134318	3.398304	0.221969
112	6	0	-6.933253	2.330120	0.026479
113	6	0	-6.063999	1.227951	-0.351588
114	6	0	-6.536061	-0.070181	-0.615559
115	6	0	-5.749871	-1.124898	-1.094435
116	6	0	-6.142076	-2.490758	-1.254347
117	6	0	-5.089682	-3.179037	-1.781766
118	6	0	-4.009873	-2.262857	-1.976828
119	6	0	-2.766400	-2.536493	-2.555154
120	6	0	-1.716302	-1.607987	-2.674224

121	6	0	-0.506889	-1.875091	-3.434764
122	6	0	0.266429	-0.777185	-3.318102
123	6	0	-0.469298	0.156734	-2.482898
124	6	0	0.039265	1.412022	-2.112100
125	1	0	0.866700	3.920018	-0.935033
126	1	0	-1.144649	5.217360	0.248765
127	1	0	-6.422645	4.404304	0.487233
128	1	0	-8.009550	2.289602	0.099150
129	1	0	-7.108279	-2.886762	-0.981371
130	1	0	-5.047382	-4.231003	-2.018823
131	1	0	-0.304756	-2.772188	-4.000204
132	1	0	1.231362	-0.592850	-3.765555
133	1	0	-3.902084	-0.192412	-1.545364
134	1	0	-2.644179	1.601250	-1.342509
135	6	0	-7.987865	-0.357619	-0.416535
136	6	0	-8.596580	-0.238308	0.831257
137	6	0	-8.789619	-0.748102	-1.497821
138	6	0	-9.955242	-0.490264	1.011551
139	1	0	-7.999984	0.059238	1.687457
140	6	0	-10.139657	-1.003764	-1.337059
141	1	0	-8.343884	-0.840082	-2.482707
142	6	0	-10.726345	-0.872855	-0.080036
143	1	0	-10.384984	-0.387032	2.000727
144	1	0	-10.757114	-1.300782	-2.177829
145	6	0	-2.565385	-3.909870	-3.105753
146	6	0	-1.579785	-4.750035	-2.580322
147	6	0	-3.353366	-4.379793	-4.160278
148	6	0	-1.389219	-6.027825	-3.091430
149	1	0	-0.958696	-4.395461	-1.763339
150	6	0	-3.160698	-5.656316	-4.674698
151	1	0	-4.110311	-3.730532	-4.589175
152	6	0	-2.179736	-6.484320	-4.140438
153	1	0	-0.621007	-6.668365	-2.670014
154	1	0	-3.773680	-6.001676	-5.501095
155	1	0	-2.028484	-7.480558	-4.543163
156	6	0	1.439503	1.757922	-2.499118
157	6	0	2.523229	1.052884	-1.969245
158	6	0	1.685931	2.794451	-3.403704
159	6	0	3.824210	1.374716	-2.335541
160	1	0	2.346498	0.255037	-1.254827
161	6	0	2.986623	3.109825	-3.778143
162	1	0	0.850229	3.343036	-3.827219
163	6	0	4.057827	2.400903	-3.244626
164	1	0	4.655314	0.829822	-1.900382
165	1	0	3.162372	3.908178	-4.492235
166	1	0	5.073890	2.649568	-3.533592
167	6	0	-3.858411	5.171494	0.607343
168	6	0	-3.620531	6.310899	-0.167031
169	6	0	-4.281768	5.337235	1.929171
170	6	0	-3.800847	7.581094	0.366839
171	1	0	-3.304757	6.196501	-1.199248
172	6	0	-4.458452	6.607010	2.464875
173	1	0	-4.468220	4.459998	2.541344
174	6	0	-4.218213	7.732675	1.684599
175	1	0	-3.619159	8.454825	-0.250783
176	1	0	-4.781320	6.717846	3.495283
177	1	0	-4.356887	8.724873	2.101677
178	8	0	-12.071973	-1.141816	-0.020475
179	6	0	-12.730036	-1.034013	1.222168

180	1	0	-12.282522	-1.705645	1.964103
181	1	0	-12.662290	-0.007879	1.609763
182	6	0	-14.183535	-1.434798	0.990655
183	1	0	-14.191292	-2.472805	0.647231
184	1	0	-14.708109	-1.387627	1.949265
185	6	0	-14.811521	-0.507337	-0.040875
186	1	0	-14.199721	-0.496449	-0.944081
187	1	0	-14.869293	0.513333	0.346493
188	7	0	-16.221672	-0.860621	-0.484192
189	6	0	-17.162426	-0.888784	0.681204
190	1	0	-16.882806	-1.696824	1.354802
191	1	0	-17.111356	0.068362	1.201197
192	1	0	-18.174304	-1.055131	0.311036
193	6	0	-16.677395	0.194608	-1.448581
194	1	0	-15.990292	0.221301	-2.294373
195	1	0	-17.682532	-0.048992	-1.793073
196	1	0	-16.682403	1.160148	-0.942705
197	6	0	-16.248697	-2.187888	-1.180538
198	1	0	-17.255839	-2.362550	-1.559498
199	1	0	-15.538089	-2.167738	-2.007073
200	1	0	-15.982205	-2.974977	-0.478098

Rotational constants (GHZ): 0.0215782 0.0061664 0.0055251

- Geometry optimization of H-dimer in the *vacuum* (WB97XD functional 6-31G(d) basis set), with BSSE correction.

```
Energy -4559,668218 Hartree
Stoichiometry    C100H88N10O2(2+)
 Framework group  C1[X(C100H88N10O2)]
 Deg. of freedom   594
 Full point group                 C1      NOp   1
 Largest Abelian subgroup         C1      NOp   1
 Largest concise Abelian subgroup C1      NOp   1
                        Standard orientation:
```

Center Number	Atomic Number	Atomic Type	Coordinates (Angstroms) X	Y	Z
1	7	0	2.101923	1.509625	1.529715
2	7	0	1.348667	-1.284775	1.777529
3	7	0	-1.448244	-0.586864	2.271851
4	7	0	-0.688422	2.209612	2.053209
5	6	0	2.186773	2.874911	1.478814
6	6	0	3.519631	3.184619	1.060873
7	6	0	4.195572	2.014746	0.901122
8	6	0	3.306266	0.937720	1.225332
9	6	0	3.608016	-0.425981	1.299439
10	6	0	2.689160	-1.437036	1.641031
11	6	0	3.097259	-2.800723	1.953832
12	6	0	1.968896	-3.469081	2.267223
13	6	0	0.878762	-2.514444	2.128575
14	6	0	-0.464338	-2.856770	2.342362
15	6	0	-1.531712	-1.949915	2.343380

16	6	0	-2.918353	-2.267408	2.474544
17	6	0	-3.618450	-1.098827	2.507789
18	6	0	-2.685767	-0.018722	2.404027
19	6	0	-2.956268	1.352731	2.500018
20	6	0	-1.990898	2.369667	2.409824
21	6	0	-2.272278	3.756372	2.758190
22	6	0	-1.115524	4.428423	2.590751
23	6	0	-0.140943	3.451216	2.127016
24	6	0	1.186845	3.790937	1.811958
25	1	0	3.887889	4.183818	0.885220
26	1	0	5.215542	1.900429	0.568891
27	1	0	4.111447	-3.173645	1.973091
28	1	0	1.872405	-4.498054	2.582043
29	1	0	-3.313865	-3.270677	2.519146
30	1	0	-4.688223	-0.986713	2.590848
31	1	0	-3.214189	4.141192	3.121821
32	1	0	-0.921900	5.474528	2.778680
33	1	0	-0.590432	-0.064180	2.133744
34	1	0	1.261362	0.989323	1.754703
35	6	0	-0.798244	-4.283641	2.625587
36	6	0	-0.491211	-5.287724	1.702626
37	6	0	-1.411178	-4.647269	3.830065
38	6	0	-0.794116	-6.618258	1.969236
39	1	0	-0.005748	-5.020668	0.771191
40	6	0	-1.716258	-5.977854	4.098319
41	1	0	-1.637472	-3.879085	4.564031
42	6	0	-1.410934	-6.967614	3.167540
43	1	0	-0.545644	-7.379616	1.235623
44	1	0	-2.184712	-6.242666	5.041593
45	1	0	-1.647066	-8.006270	3.378818
46	6	0	-4.368750	1.750528	2.767362
47	6	0	-5.031821	2.624243	1.897451
48	6	0	-5.061725	1.268093	3.883903
49	6	0	-6.354598	2.989080	2.124804
50	1	0	-4.503806	2.999454	1.026831
51	6	0	-6.385219	1.631221	4.111807
52	1	0	-4.550482	0.614215	4.584000
53	6	0	-7.037779	2.490071	3.230737
54	1	0	-6.850033	3.667498	1.436153
55	1	0	-6.901512	1.254980	4.990099
56	1	0	-8.067269	2.782948	3.416113
57	6	0	1.585981	5.228346	1.862534
58	6	0	0.962149	6.174266	1.044310
59	6	0	2.585639	5.659221	2.741889
60	6	0	1.329957	7.514034	1.095173
61	1	0	0.179253	5.855050	0.366589
62	6	0	2.955894	6.999294	2.793754
63	1	0	3.064861	4.936699	3.396466
64	6	0	2.330796	7.930730	1.968725
65	1	0	0.830625	8.229541	0.448568
66	1	0	3.728173	7.317906	3.487559
67	1	0	2.618826	8.976786	2.011303
68	6	0	5.019223	-0.824716	1.035976
69	6	0	6.082642	-0.265946	1.755137
70	6	0	5.312657	-1.770929	0.046253
71	6	0	7.399321	-0.610286	1.470850
72	1	0	5.872174	0.443521	2.549071
73	6	0	6.624387	-2.127268	-0.242011
74	1	0	4.498578	-2.206834	-0.522515

75	6	0	7.661503	-1.536836	0.468927
76	1	0	8.221458	-0.177755	2.033481
77	1	0	6.847552	-2.855441	-1.015828
78	7	0	1.456990	-0.737874	-1.979288
79	7	0	-1.337512	-1.384338	-1.406443
80	7	0	-2.079973	1.426717	-1.369723
81	7	0	0.718159	2.073677	-1.910363
82	6	0	2.625731	-0.184669	-2.427207
83	6	0	3.452358	-1.269095	-2.855484
84	6	0	2.773873	-2.431691	-2.632203
85	6	0	1.501751	-2.100797	-2.068716
86	6	0	0.486569	-3.002436	-1.722722
87	6	0	-0.825305	-2.643747	-1.376449
88	6	0	-1.847379	-3.613615	-1.000045
89	6	0	-2.988224	-2.915553	-0.842199
90	6	0	-2.657662	-1.524989	-1.124276
91	6	0	-3.614561	-0.496000	-1.140809
92	6	0	-3.324292	0.861585	-1.313936
93	6	0	-4.263801	1.928796	-1.473660
94	6	0	-3.565665	3.093074	-1.593450
95	6	0	-2.170503	2.782718	-1.522185
96	6	0	-1.112567	3.691624	-1.633361
97	6	0	0.240982	3.337952	-1.771695
98	6	0	1.317962	4.317166	-1.864011
99	6	0	2.442931	3.617303	-2.104624
100	6	0	2.047985	2.216073	-2.153943
101	6	0	2.941817	1.178454	-2.462476
102	1	0	4.432113	-1.156418	-3.295079
103	1	0	3.098139	-3.435512	-2.863361
104	1	0	-1.699730	-4.673442	-0.856889
105	1	0	-3.954066	-3.289388	-0.534437
106	1	0	-5.335741	1.804872	-1.507060
107	1	0	-3.964971	4.084285	-1.748158
108	1	0	1.220725	5.385437	-1.742189
109	1	0	3.448467	3.996716	-2.214051
110	1	0	-1.211023	0.904758	-1.350303
111	1	0	0.651205	-0.204635	-1.672085
112	6	0	-5.053580	-0.859347	-1.004377
113	6	0	-5.663162	-1.749949	-1.899356
114	6	0	-5.843461	-0.310251	0.004792
115	6	0	-7.003881	-2.080542	-1.781767
116	1	0	-5.073376	-2.182531	-2.701519
117	6	0	-7.194252	-0.625311	0.136118
118	1	0	-5.400720	0.384915	0.708107
119	6	0	-7.771251	-1.516158	-0.763197
120	1	0	-7.472039	-2.770082	-2.476927
121	1	0	-7.758322	-0.169404	0.942606
122	6	0	-1.485765	5.135569	-1.658588
123	6	0	-1.155479	5.946506	-2.750288
124	6	0	-2.179154	5.706510	-0.585077
125	6	0	-1.492930	7.295821	-2.759596
126	1	0	-0.633899	5.509787	-3.596763
127	6	0	-2.516351	7.056021	-0.593928
128	1	0	-2.421916	5.089339	0.274757
129	6	0	-2.171654	7.855741	-1.680022
130	1	0	-1.229457	7.910212	-3.615249
131	1	0	-3.040391	7.484768	0.255214
132	1	0	-2.432547	8.909749	-1.687255
133	6	0	4.322820	1.537198	-2.899299

134	6	0	4.528192	2.340185	-4.026965
135	6	0	5.441316	1.074572	-2.199043
136	6	0	5.813949	2.680844	-4.434240
137	1	0	3.668316	2.694543	-4.587419
138	6	0	6.727057	1.414774	-2.604682
139	1	0	5.296637	0.445416	-1.328552
140	6	0	6.919227	2.221887	-3.722346
141	1	0	5.952244	3.303683	-5.312770
142	1	0	7.578900	1.052906	-2.034577
143	1	0	7.921812	2.493247	-4.040005
144	6	0	0.842928	-4.449563	-1.785899
145	6	0	1.875478	-4.959434	-0.990678
146	6	0	0.158163	-5.324642	-2.636425
147	6	0	2.202042	-6.311016	-1.030399
148	1	0	2.397666	-4.290706	-0.313035
149	6	0	0.485270	-6.675991	-2.677516
150	1	0	-0.632790	-4.935526	-3.270671
151	6	0	1.506319	-7.174358	-1.872185
152	1	0	2.995168	-6.691619	-0.393345
153	1	0	-0.055272	-7.340478	-3.344935
154	1	0	1.759285	-8.229925	-1.903162
155	8	0	8.974131	-1.892625	0.190063
156	8	0	-9.092424	-1.898181	-0.731225
157	6	0	-9.924493	-1.326776	0.247549
158	1	0	-9.936603	-0.230010	0.160824
159	1	0	-9.583636	-1.582153	1.258804
160	6	0	-11.320505	-1.903192	0.022059
161	1	0	-11.991654	-1.503927	0.788584
162	1	0	-11.254712	-2.987556	0.151862
163	6	0	-11.788631	-1.536071	-1.380273
164	1	0	-11.008354	-1.784484	-2.102776
165	1	0	-11.990880	-0.462926	-1.445597
166	6	0	9.582544	-1.090782	-0.801308
167	1	0	9.039259	-1.161854	-1.752385
168	1	0	9.585236	-0.033500	-0.494826
169	6	0	11.006530	-1.610721	-0.982039
170	1	0	11.502310	-1.022755	-1.760462
171	1	0	10.935697	-2.647519	-1.324406
172	6	0	11.738476	-1.513180	0.350235
173	1	0	11.113283	-1.936125	1.139463
174	1	0	11.946127	-0.467385	0.595381
175	7	0	-13.051906	-2.228732	-1.859979
176	7	0	13.073102	-2.232985	0.428300
177	6	0	-12.824896	-3.701998	-2.014702
178	1	0	-13.724266	-4.151947	-2.437242
179	1	0	-11.977347	-3.860348	-2.682624
180	1	0	-12.618923	-4.145245	-1.041863
181	6	0	-13.410313	-1.656769	-3.198995
182	1	0	-12.574169	-1.807896	-3.882860
183	1	0	-14.298272	-2.164446	-3.577965
184	1	0	-13.611544	-0.590574	-3.088071
185	6	0	-14.184583	-1.993708	-0.910192
186	1	0	-13.964028	-2.475257	0.041828
187	1	0	-14.308943	-0.919276	-0.766578
188	1	0	-15.094698	-2.419910	-1.334567
189	6	0	13.997978	-1.762545	-0.650151
190	1	0	13.595899	-2.048738	-1.621386
191	1	0	14.090496	-0.677122	-0.588393
192	1	0	14.974034	-2.227820	-0.505563

193	6	0	13.683469	-1.928321	1.763443
194	1	0	12.997331	-2.251734	2.547206
195	1	0	14.630382	-2.462260	1.853539
196	1	0	13.854970	-0.853878	1.839345
197	6	0	12.884585	-3.715566	0.316397
198	1	0	13.849714	-4.202712	0.461602
199	1	0	12.180720	-4.040840	1.083433
200	1	0	12.497417	-3.961897	-0.670852

Rotational constants (GHZ): 0.0209211 0.0089945
0.0074476

- Geometry optimization of J-dimer in the *vacuum* (WB97XD functional 6-31G(d) basis set), with BSSE correction.

```
Energy -4559,661941 Hartree
Stoichiometry    C100H88N10O2(2+)
 Framework group  C1[X(C100H88N10O2)]
 Deg. of freedom   594
 Full point group                 C1      NOp   1
 Largest Abelian subgroup         C1      NOp   1
 Largest concise Abelian subgroup C1      NOp   1
                        Standard orientation:
```

Center Number	Atomic Number	Atomic Type	Coordinates (Angstroms) X	Y	Z
1	7	0	-3.805397	1.549968	-0.805415
2	7	0	-3.480560	-1.302288	-0.983138
3	7	0	-0.602196	-1.048103	-1.383447
4	7	0	-0.953049	1.812054	-1.351192
5	6	0	-3.660092	2.897995	-0.612079
6	6	0	-4.955878	3.403818	-0.270397
7	6	0	-5.835286	2.363574	-0.300979
8	6	0	-5.111512	1.177237	-0.655412
9	6	0	-5.623880	-0.113605	-0.815209
10	6	0	-4.838867	-1.266597	-1.004283
11	6	0	-5.392801	-2.601239	-1.175840
12	6	0	-4.341026	-3.446641	-1.196765
13	6	0	-3.149233	-2.620441	-1.067695
14	6	0	-1.850367	-3.151758	-1.066209
15	6	0	-0.674082	-2.401426	-1.199550
16	6	0	0.665744	-2.894074	-1.275609
17	6	0	1.489681	-1.842112	-1.549029
18	6	0	0.685720	-0.664429	-1.632591
19	6	0	1.123011	0.634675	-1.931397
20	6	0	0.332363	1.785299	-1.801642
21	6	0	0.815045	3.132891	-2.077347
22	6	0	-0.178299	3.968798	-1.713599
23	6	0	-1.277899	3.124558	-1.271792
24	6	0	-2.508197	3.646849	-0.835601
25	1	0	-5.165625	4.439663	-0.046665
26	1	0	-6.893471	2.396746	-0.087729
27	1	0	-6.439266	-2.848695	-1.283516
28	1	0	-4.355854	-4.519417	-1.324562
29	1	0	0.957869	-3.921938	-1.127807
30	1	0	2.564816	-1.861381	-1.643820
31	1	0	1.779854	3.391775	-2.488008

32	1	0	-0.192701	5.048489	-1.761475
33	1	0	-1.390316	-0.412277	-1.342067
34	1	0	-3.046530	0.916438	-1.029808
35	6	0	-1.717966	-4.633153	-0.965445
36	6	0	-2.232421	-5.304595	0.149559
37	6	0	-1.118167	-5.382851	-1.982523
38	6	0	-2.158590	-6.689690	0.241331
39	1	0	-2.689130	-4.726299	0.946657
40	6	0	-1.038377	-6.768955	-1.888805
41	1	0	-0.729169	-4.873645	-2.859357
42	6	0	-1.564217	-7.426063	-0.780035
43	1	0	-2.559496	-7.194483	1.115096
44	1	0	-0.576370	-7.336893	-2.690611
45	1	0	-1.510177	-8.508648	-0.711953
46	6	0	2.516071	0.758565	-2.450359
47	6	0	3.466823	1.572243	-1.828434
48	6	0	2.887272	0.057662	-3.604972
49	6	0	4.750263	1.689672	-2.350531
50	1	0	3.195888	2.112657	-0.928443
51	6	0	4.171455	0.171013	-4.125542
52	1	0	2.154199	-0.570078	-4.102780
53	6	0	5.106548	0.991392	-3.500056
54	1	0	5.472610	2.321916	-1.844689
55	1	0	4.435633	-0.372845	-5.027763
56	1	0	6.108729	1.088321	-3.908541
57	6	0	-2.617663	5.124713	-0.632211
58	6	0	-2.584748	5.659573	0.657724
59	6	0	-2.749899	5.990469	-1.721132
60	6	0	-2.672399	7.034399	0.855865
61	1	0	-2.485623	4.989815	1.506219
62	6	0	-2.834099	7.365516	-1.524240
63	1	0	-2.781058	5.577894	-2.725703
64	6	0	-2.794023	7.890581	-0.235008
65	1	0	-2.640330	7.436657	1.863989
66	1	0	-2.933915	8.027489	-2.379320
67	1	0	-2.859198	8.963479	-0.081297
68	6	0	-7.106869	-0.258493	-0.750707
69	6	0	-7.924629	0.417971	-1.664151
70	6	0	-7.719502	-1.069300	0.212161
71	6	0	-9.308549	0.296588	-1.616502
72	1	0	-7.463818	1.037428	-2.427296
73	6	0	-9.102692	-1.199804	0.268660
74	1	0	-7.102422	-1.610816	0.922256
75	6	0	-9.889047	-0.514506	-0.648574
76	1	0	-9.936739	0.811213	-2.337881
77	1	0	-9.574635	-1.833443	1.013114
78	7	0	0.722746	-1.547903	1.930307
79	7	0	3.537251	-1.656066	1.274342
80	7	0	3.829260	1.243938	1.461565
81	7	0	0.988636	1.377146	1.942975
82	6	0	-0.538294	-1.204500	2.332950
83	6	0	-1.204658	-2.423223	2.680786
84	6	0	-0.325650	-3.449777	2.505166
85	6	0	0.907554	-2.899461	2.030928
86	6	0	2.084933	-3.593736	1.741962
87	6	0	3.282395	-2.990541	1.321215
88	6	0	4.435879	-3.760747	0.874580
89	6	0	5.397292	-2.862190	0.577977
90	6	0	4.826235	-1.551072	0.854515

91	6	0	5.569657	-0.365464	0.771123
92	6	0	5.100258	0.912874	1.080756
93	6	0	5.826893	2.139286	0.967440
94	6	0	4.973452	3.165716	1.250548
95	6	0	3.691934	2.604046	1.546473
96	6	0	2.501331	3.306670	1.753852
97	6	0	1.239456	2.713482	1.907666
98	6	0	0.024638	3.486350	2.129357
99	6	0	-0.959394	2.587387	2.319289
100	6	0	-0.336558	1.273639	2.220224
101	6	0	-1.050923	0.088425	2.454791
102	1	0	-2.221217	-2.478790	3.041345
103	1	0	-0.492327	-4.499976	2.692975
104	1	0	4.483647	-4.837606	0.797465
105	1	0	6.400201	-3.055880	0.224344
106	1	0	6.864986	2.211641	0.677214
107	1	0	5.186486	4.224441	1.233474
108	1	0	-0.044420	4.564401	2.135147
109	1	0	-2.009433	2.768978	2.498977
110	1	0	3.068885	0.583564	1.590405
111	1	0	1.445865	-0.889909	1.659763
112	6	0	6.989445	-0.455843	0.315899
113	6	0	8.046442	-0.302203	1.220437
114	6	0	7.294784	-0.698415	-1.021671
115	6	0	9.365007	-0.393337	0.802153
116	1	0	7.825264	-0.115977	2.267028
117	6	0	8.615482	-0.796236	-1.459526
118	1	0	6.485222	-0.807856	-1.736660
119	6	0	9.648516	-0.644801	-0.539398
120	1	0	10.184685	-0.285672	1.505274
121	1	0	8.809688	-0.988227	-2.509168
122	6	0	2.603873	4.796139	1.798622
123	6	0	3.272679	5.430838	2.849304
124	6	0	2.030777	5.577721	0.791869
125	6	0	3.363533	6.818880	2.893181
126	1	0	3.713742	4.828814	3.638867
127	6	0	2.116505	6.965239	0.838212
128	1	0	1.510492	5.084260	-0.022682
129	6	0	2.784329	7.589580	1.888557
130	1	0	3.880316	7.299164	3.718789
131	1	0	1.660173	7.559111	0.051675
132	1	0	2.851145	8.672755	1.925899
133	6	0	-2.444381	0.218574	2.979705
134	6	0	-2.649537	0.729370	4.266190
135	6	0	-3.548222	-0.148033	2.209636
136	6	0	-3.936006	0.876701	4.772143
137	1	0	-1.790154	1.013535	4.866641
138	6	0	-4.835735	0.008246	2.715492
139	1	0	-3.392354	-0.539046	1.208590
140	6	0	-5.034364	0.518554	3.994218
141	1	0	-4.080185	1.272476	5.773094
142	1	0	-5.687053	-0.255660	2.096792
143	1	0	-6.040752	0.640025	4.384653
144	6	0	2.084609	-5.072706	1.947907
145	6	0	1.263010	-5.925529	1.205807
146	6	0	2.933579	-5.629414	2.912481
147	6	0	1.297594	-7.300815	1.417359
148	1	0	0.588453	-5.515183	0.460909
149	6	0	2.966226	-7.002684	3.125009

150	1	0	3.567804	-4.971449	3.499403
151	6	0	2.148725	-7.843811	2.374429
152	1	0	0.654955	-7.944805	0.826312
153	1	0	3.627674	-7.415616	3.880801
154	1	0	2.173442	-8.917125	2.537383
155	8	0	-11.268096	-0.666835	-0.610855
156	8	0	10.984765	-0.728116	-0.857252
157	6	0	11.338329	-1.005954	-2.188984
158	1	0	10.980492	-0.214614	-2.864713
159	1	0	10.908803	-1.958219	-2.524721
160	6	0	12.862257	-1.100539	-2.223293
161	1	0	13.174993	-1.337456	-3.244819
162	1	0	13.154550	-1.929481	-1.571907
163	6	0	13.455893	0.218736	-1.746603
164	1	0	12.981391	0.516152	-0.809223
165	1	0	13.281858	1.005148	-2.486918
166	6	0	-11.937959	0.338124	0.122845
167	1	0	-11.602249	0.349634	1.167483
168	1	0	-11.736295	1.331456	-0.305243
169	6	0	-13.427830	0.009619	0.065295
170	1	0	-13.978605	0.757582	0.643749
171	1	0	-13.566357	-0.965885	0.541132
172	6	0	-13.871248	-0.013699	-1.391763
173	1	0	-13.166643	-0.609006	-1.976432
174	1	0	-13.891060	1.000930	-1.800415
175	7	0	14.951880	0.214836	-1.485308
176	7	0	-15.247950	-0.597237	-1.656234
177	6	0	15.288639	-0.683192	-0.333544
178	1	0	16.355131	-0.595482	-0.121983
179	1	0	14.707356	-0.374930	0.536304
180	1	0	15.052512	-1.714220	-0.591415
181	6	0	15.361633	1.614312	-1.136185
182	1	0	14.794443	1.941044	-0.263719
183	1	0	16.429307	1.628639	-0.913278
184	1	0	15.150615	2.268490	-1.982998
185	6	0	15.706448	-0.225450	-2.700633
186	1	0	15.472952	-1.268027	-2.913563
187	1	0	15.416839	0.403158	-3.544134
188	1	0	16.775264	-0.121097	-2.508806
189	6	0	-16.292350	0.112845	-0.853306
190	1	0	-16.120531	-0.071697	0.206558
191	1	0	-16.232280	1.182253	-1.061372
192	1	0	-17.274685	-0.266932	-1.137688
193	6	0	-15.549741	-0.423758	-3.114458
194	1	0	-14.773950	-0.922197	-3.697016
195	1	0	-16.522372	-0.866544	-3.333075
196	1	0	-15.565607	0.641005	-3.350251
197	6	0	-15.275736	-2.060681	-1.333836
198	1	0	-16.250321	-2.462056	-1.614822
199	1	0	-14.487021	-2.561208	-1.896812
200	1	0	-15.117310	-2.203901	-0.266330

Rotational constants (GHZ): 0.0232149 0.0069393 0.0059722

- Geometry optimization of cation-π dimer in the *vacuum* (WB97XD functional 6-31G(d) basis set), with BSSE correction.

Energy -4559,687595 Hartree
Stoichiometry   C100H88N10O2(2+)
Framework group C1[X(C100H88N10O2)]
Deg. of freedom   594
Full point group   C1   NOp   1
Largest Abelian subgroup   C1   NOp   1
Largest concise Abelian subgroup C1   NOp   1

Standard orientation:

Center Number	Atomic Number	Atomic Type	Coordinates (Angstroms) X	Y	Z
1	7	0	-4.229825	-3.402268	1.406209
2	7	0	-3.337345	-0.839200	2.509499
3	7	0	-4.862799	0.644256	0.532476
4	7	0	-5.573593	-1.903180	-0.686441
5	6	0	-4.787540	-4.445673	0.724738
6	6	0	-4.425899	-5.638715	1.426188
7	6	0	-3.672422	-5.280217	2.505028
8	6	0	-3.530884	-3.857017	2.490493
9	6	0	-2.805186	-3.081542	3.397084
10	6	0	-2.713432	-1.681875	3.378545
11	6	0	-1.872265	-0.944183	4.303225
12	6	0	-1.989489	0.359498	3.960719
13	6	0	-2.920108	0.407986	2.841621
14	6	0	-3.313278	1.603567	2.203078
15	6	0	-4.247964	1.686956	1.171861
16	6	0	-4.706592	2.885489	0.536647
17	6	0	-5.533806	2.534790	-0.484496
18	6	0	-5.618917	1.104319	-0.512242
19	6	0	-6.239109	0.330024	-1.495140
20	6	0	-6.223921	-1.075764	-1.545588
21	6	0	-6.990502	-1.830095	-2.530454
22	6	0	-6.799710	-3.131104	-2.238762
23	6	0	-5.913838	-3.163889	-1.081468
24	6	0	-5.550947	-4.357928	-0.446031
25	1	0	-4.718591	-6.635649	1.132123
26	1	0	-3.247211	-5.930766	3.255266
27	1	0	-1.285817	-1.373116	5.104341
28	1	0	-1.531004	1.206705	4.451108
29	1	0	-4.422485	3.884344	0.830311
30	1	0	-6.016238	3.194933	-1.188907
31	1	0	-7.614668	-1.405921	-3.303601
32	1	0	-7.233859	-3.992685	-2.725027
33	1	0	-4.701420	-0.334791	0.740608
34	1	0	-4.316801	-2.429098	1.135218
35	6	0	-2.554637	2.849015	2.525291
36	6	0	-2.489271	3.412404	3.805352
37	6	0	-1.779717	3.423928	1.512327
38	6	0	-1.582527	4.430151	4.089614
39	1	0	-3.131133	3.027930	4.592359
40	6	0	-0.884528	4.450870	1.777901
41	1	0	-1.839223	3.015665	0.509879
42	6	0	-0.741212	4.904699	3.083401
43	1	0	-1.506306	4.841781	5.091534
44	1	0	-0.255243	4.861019	0.993479
45	6	0	-6.919793	1.074067	-2.596853
46	6	0	-8.039201	1.873230	-2.347841
47	6	0	-6.425279	0.992449	-3.902432

48	6	0	-8.647455	2.579254	-3.381124
49	1	0	-8.436543	1.932924	-1.338586
50	6	0	-7.038674	1.690593	-4.937388
51	1	0	-5.554851	0.373327	-4.102113
52	6	0	-8.149279	2.489325	-4.678364
53	1	0	-9.520097	3.191035	-3.174005
54	1	0	-6.647874	1.611454	-5.947695
55	1	0	-8.630234	3.033373	-5.485445
56	6	0	-6.023178	-5.648825	-1.031037
57	6	0	-6.969107	-6.430935	-0.361912
58	6	0	-5.524942	-6.093690	-2.258727
59	6	0	-7.402778	-7.635240	-0.907290
60	1	0	-7.371070	-6.084957	0.586136
61	6	0	-5.958866	-7.297322	-2.805055
62	1	0	-4.798745	-5.482599	-2.786503
63	6	0	-6.897517	-8.072042	-2.128936
64	1	0	-8.142668	-8.229457	-0.380015
65	1	0	-5.564501	-7.630071	-3.760454
66	1	0	-7.238333	-9.010450	-2.555044
67	6	0	-2.060157	-3.822434	4.460042
68	6	0	-0.931626	-4.580888	4.135854
69	6	0	-2.479298	-3.770207	5.792283
70	6	0	-0.236367	-5.274298	5.121773
71	1	0	-0.602443	-4.625243	3.100998
72	6	0	-1.784062	-4.462394	6.779305
73	1	0	-3.359820	-3.188279	6.048664
74	6	0	-0.661525	-5.216120	6.446447
75	1	0	0.635871	-5.863945	4.855089
76	1	0	-2.125558	-4.419732	7.808976
77	1	0	-0.123975	-5.761104	7.216274
78	8	0	0.279699	5.782731	3.363308
79	6	0	1.388480	5.184641	4.018055
80	1	0	2.118415	5.990348	4.112651
81	1	0	1.107757	4.865678	5.032645
82	6	0	2.027243	4.013674	3.241850
83	1	0	1.892748	4.199737	2.171700
84	1	0	3.104328	4.036066	3.434739
85	6	0	1.464744	2.649066	3.649033
86	1	0	0.373274	2.650563	3.669478
87	1	0	1.825117	2.381301	4.647199
88	7	0	1.852857	1.503839	2.733964
89	6	0	3.313224	1.529235	2.428577
90	1	0	3.553929	2.431603	1.870943
91	1	0	3.869864	1.505011	3.367622
92	1	0	3.553883	0.657380	1.819182
93	6	0	1.533500	0.208712	3.409845
94	1	0	0.469497	0.179232	3.648605
95	1	0	1.769689	-0.604091	2.721145
96	1	0	2.134214	0.120105	4.316779
97	6	0	1.063614	1.564664	1.458726
98	1	0	1.430666	0.788784	0.782963
99	1	0	0.011526	1.407003	1.699855
100	1	0	1.191163	2.534743	0.979278
101	7	0	-0.330769	1.077380	-1.579788
102	7	0	2.212352	-0.240864	-1.041294
103	7	0	3.577405	2.309368	-0.786615
104	7	0	1.017357	3.602934	-1.246668
105	6	0	-1.379282	1.922071	-1.831923
106	6	0	-2.563389	1.120482	-1.804152

107	6	0	-2.205747	-0.165800	-1.535114
108	6	0	-0.778366	-0.208534	-1.424664
109	6	0	0.017089	-1.346708	-1.260573
110	6	0	1.420664	-1.340005	-1.181398
111	6	0	2.222545	-2.547800	-1.294478
112	6	0	3.512893	-2.153696	-1.234772
113	6	0	3.492855	-0.710019	-1.045838
114	6	0	4.641600	0.069475	-0.835293
115	6	0	4.638104	1.458673	-0.625839
116	6	0	5.706293	2.242046	-0.091928
117	6	0	5.257770	3.524410	0.069173
118	6	0	3.910108	3.586263	-0.401161
119	6	0	3.090134	4.718041	-0.504374
120	6	0	1.782714	4.707339	-1.023681
121	6	0	1.091187	5.918183	-1.434390
122	6	0	-0.105011	5.522035	-1.918387
123	6	0	-0.155822	4.077880	-1.753553
124	6	0	-1.311344	3.311170	-1.999514
125	1	0	-3.561896	1.504452	-1.937242
126	1	0	-2.868716	-1.009920	-1.404524
127	1	0	1.834540	-3.545698	-1.442219
128	1	0	4.395941	-2.769389	-1.328433
129	1	0	6.681616	1.855449	0.165637
130	1	0	5.806627	4.362473	0.472536
131	1	0	1.496144	6.919235	-1.400158
132	1	0	-0.876833	6.135569	-2.359100
133	1	0	2.669165	2.044794	-1.150201
134	1	0	0.641353	1.357265	-1.548335
135	6	0	5.977169	-0.594355	-0.803785
136	6	0	6.291511	-1.586779	0.124006
137	6	0	6.965363	-0.224073	-1.727705
138	6	0	7.538932	-2.210835	0.134389
139	1	0	5.547706	-1.886573	0.856339
140	6	0	8.210379	-0.829762	-1.729704
141	1	0	6.744203	0.545380	-2.461005
142	6	0	8.498189	-1.830099	-0.800341
143	1	0	7.738661	-2.976586	0.875600
144	1	0	8.969611	-0.543537	-2.449999
145	6	0	3.651238	6.025726	-0.059583
146	6	0	2.990029	6.763632	0.930519
147	6	0	4.821665	6.549380	-0.619709
148	6	0	3.497426	7.984038	1.362548
149	1	0	2.062759	6.388225	1.355492
150	6	0	5.327725	7.771167	-0.187931
151	1	0	5.323066	6.004236	-1.414297
152	6	0	4.670521	8.488873	0.807861
153	1	0	2.969847	8.544663	2.128191
154	1	0	6.230516	8.169740	-0.640248
155	1	0	5.064342	9.443852	1.141078
156	6	0	-2.582724	4.002768	-2.356978
157	6	0	-3.090630	5.027889	-1.549550
158	6	0	-3.317761	3.610205	-3.481551
159	6	0	-4.294416	5.648326	-1.862995
160	1	0	-2.546928	5.325662	-0.657677
161	6	0	-4.529676	4.219506	-3.786288
162	1	0	-2.932856	2.821958	-4.122025
163	6	0	-5.019575	5.242922	-2.980056
164	1	0	-4.673141	6.440571	-1.224350
165	1	0	-5.095606	3.891154	-4.651423

166	1	0	-5.965080	5.718834	-3.220479
167	6	0	-0.690666	-2.659688	-1.240007
168	6	0	-1.386953	-3.106193	-2.367284
169	6	0	-0.673088	-3.459670	-0.094018
170	6	0	-2.055903	-4.324513	-2.346258
171	1	0	-1.398463	-2.492875	-3.263618
172	6	0	-1.342910	-4.677602	-0.075077
173	1	0	-0.147121	-3.111587	0.790921
174	6	0	-2.039582	-5.111110	-1.198737
175	1	0	-2.588882	-4.657950	-3.231342
176	1	0	-1.346294	-5.281328	0.826323
177	1	0	-2.580139	-6.052263	-1.175001
178	8	0	9.752354	-2.375657	-0.888222
179	6	0	10.097701	-3.417214	-0.004946
180	1	0	10.052148	-3.081838	1.038725
181	1	0	9.411542	-4.269131	-0.120570
182	6	0	11.530484	-3.824738	-0.339907
183	1	0	12.170966	-2.953104	-0.175973
184	1	0	11.833976	-4.611172	0.357838
185	6	0	11.598568	-4.300979	-1.785706
186	1	0	11.162702	-3.545725	-2.443169
187	1	0	11.033995	-5.230148	-1.907644
188	7	0	12.989254	-4.585091	-2.324504
189	6	0	13.692420	-5.607272	-1.485617
190	1	0	13.879514	-5.196775	-0.494046
191	1	0	13.065710	-6.497381	-1.412019
192	1	0	14.641231	-5.859752	-1.960928
193	6	0	12.841424	-5.118757	-3.719001
194	1	0	12.315609	-4.380990	-4.326332
195	1	0	13.831789	-5.305995	-4.135855
196	1	0	12.271648	-6.048196	-3.685832
197	6	0	13.809012	-3.331213	-2.376454
198	1	0	14.764608	-3.557668	-2.851157
199	1	0	13.271557	-2.581223	-2.958128
200	1	0	13.983728	-2.965371	-1.365907

Rotational constants (GHZ): 0.0159705 0.0099016 0.0078173
